# An approximate line attractor in the hypothalamus encodes an aggressive state

**DOI:** 10.1016/j.cell.2022.11.027

**Published:** 2023-01-05

**Authors:** Aditya Nair, Tomomi Karigo, Bin Yang, Surya Ganguli, Mark J. Schnitzer, Scott W. Linderman, David J. Anderson, Ann Kennedy

**Affiliations:** 1 - Division of Biology and Biological Engineering, Caltech, Pasadena, CA 91125, USA; 2 - Howard Hughes Medical Institute; 3 - Tianqiao and Chrissy Chen Institute for Neuroscience, Caltech, Pasadena, CA 91125, USA; 4 - Department of Applied Physics, Stanford University, Stanford, CA, USA; 5 - Department of Biology, Stanford University, Stanford, CA, USA; 6 - Department of Statistics, Stanford University, Stanford, CA 94305, USA; 7 - Wu Tsai Neurosciences Institute, Stanford University, Stanford, CA 94305, USA; 8 - Department of Neuroscience, Feinberg School of Medicine, Northwestern University, Chicago IL 60611, USA; 9 - Present address: Kennedy Krieger Institute, Solomon H. Snyder Department of Neuroscience, Johns Hopkins University School of Medicine, Baltimore, MD 21205 USA; 10 - Present address: Solomon H. Snyder Department of Neuroscience, Johns Hopkins University School of Medicine, Baltimore, MD 21205 USA; 11 - Lead contact

## Abstract

The hypothalamus regulates innate social behaviors, including mating and aggression. These behaviors can be evoked by optogenetic stimulation of specific neuronal subpopulations within MPOA and VMHvl, respectively. Here we perform dynamical systems modeling of population neuronal activity in these nuclei during social behaviors. In VMHvl, unsupervised analysis identified a dominant dimension of neural activity with a large time constant (>50s), generating an approximate line attractor in neural state space. Progression of the neural trajectory along this attractor was correlated with an escalation of agonistic behavior, suggesting that it may encode a scalable state of aggressiveness. Consistent with this, individual differences in the magnitude of the attractor time constant were strongly correlated with differences in aggressiveness. In contrast, line attractors were not observed in MPOA during mating; instead, neurons with fast dynamics were tuned to specific actions. Thus, different hypothalamic nuclei employ distinct neural population codes to represent similar social behaviors.

## In Brief

Different hypothalamic regions utilize distinct neural dynamics to encode mating and aggressive behaviors.

## Graphical Abstract

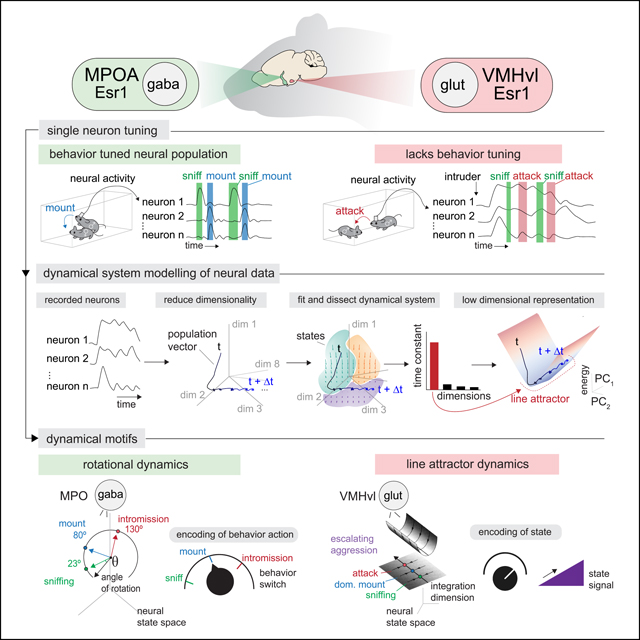

## Introduction

A fundamental problem in neuroscience is to understand how the brain controls innate behaviors. Many such behaviors are governed by the hypothalamus, a deep subcortical brain region present in all vertebrates^1,2^. Classical brain stimulation and lesion experiments have implicated different hypothalamic regions (“nuclei”) in diverse innate behaviors (reviewed in^3–9^). More recently, optogenetic stimulation has identified genetically marked neuronal subpopulations that can evoke such behaviors^10–13^,(reviewed in^14–17^). Genetic ablation or reversible silencing has demonstrated that these subpopulations are essential for natural occurrences of these behaviors^10–12,18^.

An important open question is how the activity of these neural subpopulations during naturally occurring behavior reflects their “causative” function. Relatively few single unit recordings have been performed in hypothalamic nuclei because of their inaccessibility^13,19–21^. Recordings of bulk calcium signals^22^ have confirmed that these neuronal subpopulations are active during the natural behaviors they can artificially evoke^23–25^. However, this averaging method obscures individual cell activity patterns.

**Figure 1:**
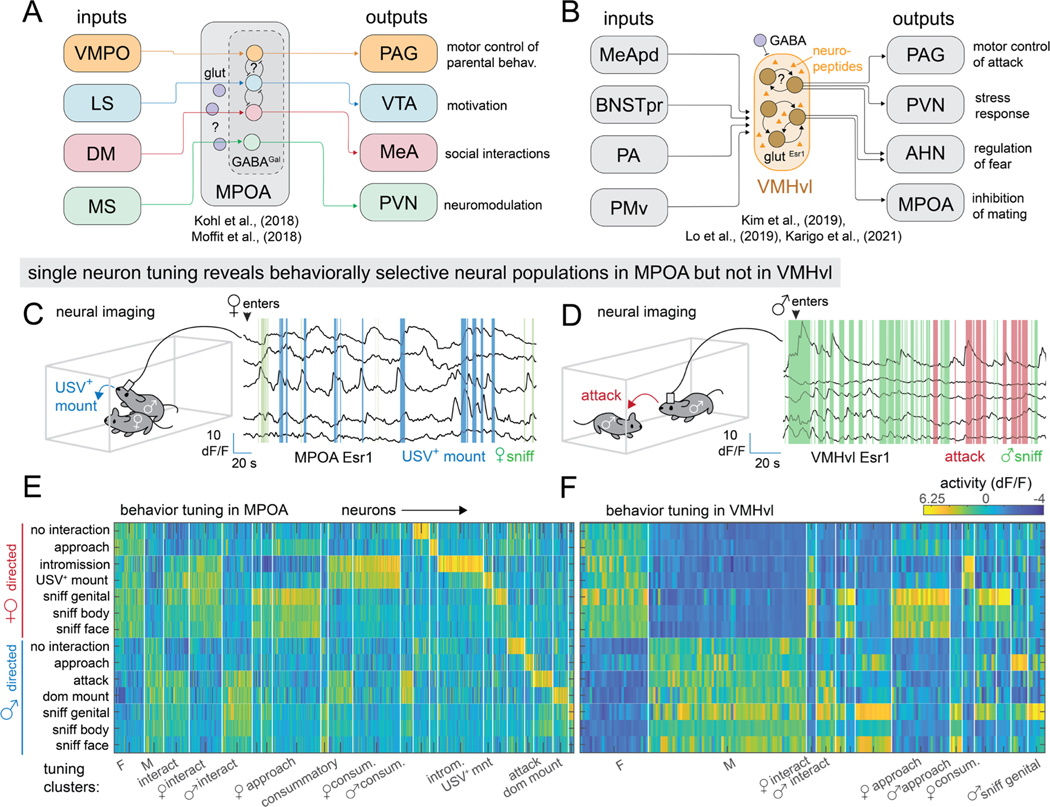
Cytoarchitectures and cellular representations in a neural system regulating social behavior A, B: cytoarchitecture of MPOA (A) and VMHvl (B). C, D: example traces from Esr1 ^+^ neurons in MPOA (C) and VMHvl (D). E, F: clustering of recorded Esr1^+^ neurons in MPOA (E, n =306 neurons from 3 mice) and VMHvl (F, n = 391 neurons from 4 mice) using a regression model. Rows, hand-annotated behaviors; columns, individual neurons.

Miniature head-mounted microscopes allow calcium imaging with single-cell resolution in freely moving animals^26,27^. Application of this approach to the hypothalamus has identified cells exhibiting stimulus-locked activity during natural behavior^28–30^. For example, imaging of estrogen receptor type 1 (Esr1)-expressing neurons in the medial preoptic area (MPOA), whose optogenetic activation can elicit mounting behavior in male mice^31,32^, has revealed cells that respond specifically during spontaneous mounting of females (see also Fig. 1E). Such results, together with single-cell transcriptomic analysis, have reinforced the prevailing view that the hypothalamus controls different survival behaviors via genetically determined, functionally specific neuronal subpopulations^33,34^.

The case of aggression, however, presents a paradox seemingly at odds with this view. On the one hand, optogenetic stimulation of Esr1^+^ neurons in the ventrolateral subdivision of the ventromedial hypothalamus (VMHvl) neurons triggers attack behavior^12,35–37^, identifying these neurons as the likely cellular substrate of electrical brain-stimulated aggression^4,7,38^. Conversely, genetic ablation of VMHvl neurons expressing the progesterone receptor (PR; co-expressed with Esr1) or optogenetic silencing of VMHvl^Esr1^ neurons blocks natural aggression^18^,^12^

On the other hand, miniscope imaging of VMHvl^Esr1^ neurons during natural fighting revealed surprisingly few cells that exhibited time-locked, attack-specific activity^29^. Instead most such neurons exhibited “mixed selectivity,” responding during different phases of an aggressive interaction. Different subsets of Esr1^+^ neurons responded to male vs. female conspecifics, suggesting an encoding of conspecific sex^29,31,39^. Nevertheless, decoders trained on VMHvl^Esr1^ neural imaging data could accurately distinguish episodes of attack from sniffing^29^.

Thus observational vs. perturbational studies of VMHvl^Esr1^ neurons yield seemingly inconsistent views: these neurons causally control aggressive behavior, yet very few of them are specifically “tuned” to attack. There are two possible explanations for this paradox. First, the small fraction of VMHvl^Esr1^ neurons that are more active during attack may be the ones responsible for the specific causative influence of this population. Alternatively, the majority of VMHvl^Esr1^ neurons, despite their mixed behavioral selectivity, may control attack through some type of population code.

In other systems where there is no clear correlation between single-unit spiking patterns and behavior, modeling neural populations as a dynamical system^40–42^ (reviewed in^43^) has revealed signals in the dynamics of population activity that can robustly predict motor actions^44,45^. We have therefore carried out similar modeling of VMHvl^Esr1^ neural activity dynamics during naturalistic social behaviors, using legacy data from previous studies^29,31,39^. Our results reveal line attractor dynamics in VMHvl that correlate with escalating levels of aggressive behavior, suggesting they may represent or encode an aggressive internal state. Strikingly, line attractor dynamics are absent in MPOA activity during both mating and aggression. This analysis therefore reveals fundamental differences in the neural coding of social behaviors by different hypothalamic nuclei.

## Results

### Cellular tuning analysis confirms behaviorally selective neural populations in MPOA but not in VMHvl

Calcium imaging of MPOA^Esr1^ or VMHvl^Esr1^ neurons revealed distinct patterns of neuronal activation during social interactions^29,31^ (Figure 1A, B). To quantify these differences, we re-analyzed calcium imaging data^31^ from sexually experienced male C57Bl/6N^*Esr1−2A-Cre/+*^ mice during standard resident-intruder assays, using male or female BalbC intruders (Figure 1C, D). We then computed the mean activity of each neuron during each of 14 different hand-annotated actions and clustered them using a regression model (VMHvl: N= 306 neurons from 3 mice; MPOA: N= 391 neurons from 4 mice, see Methods).

Confirming previous observations^29,31^, many MPOA clusters contained neurons only active during specific behavioral actions, such as intromission or mounting towards females (Figure 1E). In contrast, most VMHvl^Esr1^ neurons were activated in response to either males or females, with very few neurons showing behavior-specific activation (Figure 1F).

### Unsupervised dynamical systems analysis of neural activity during social behavior

In other systems, population analysis via fit dynamical systems has revealed a neural encoding of behavioral actions that were not apparent in neuron-by-neuron analysis^43,44,46,47^. We therefore investigated whether behavioral representations among VMHvl^Esr1^ neurons might be encoded at a population level, using an unsupervised dynamical systems approach.

To do so, we fit a dynamical model to the population activity of VMHvl^Esr1^ cells from each of multiple mice (n=6), from two different studies^31,39^ in which recordings were made throughout male-male or male-female encounters (average duration 5.1± 0.68 min and 11.4± 0.68 min, respectively; mean ± SEM). Specifically, we fit a recurrent switching linear dynamical system (rSLDS) model^48^, which approximates a complex non-linear dynamical system as a composite of more easily interpretable linear dynamical systems, or “states” (Supplemental Figure S1A).

**Figure 2:**
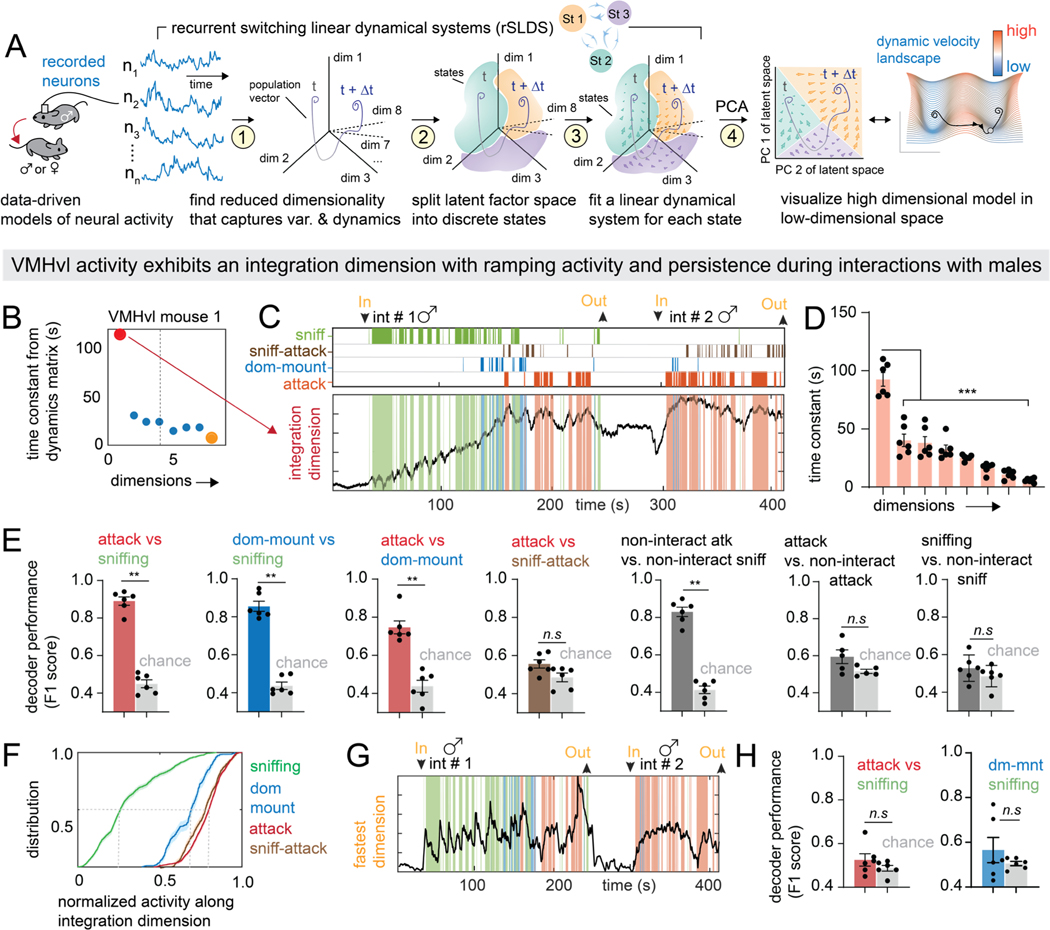
Dynamical analysis of VMHvl neural activity reveals an integrator dimension that correlates with aggressive escalation A: schematic illustrating rSLDS analysis. B: time constants of rSLDS dimensions (see A➀) in attack enriched state from VMHvl mouse 1. Dimensions with longest (red dot) and shortest (yellow dot) time constants are indicated. C: projection onto time axis of integration dimension with overlayed behavior annotations. D: average time constant of all dimensions, arranged in decreasing order. (***p < 0.001, n = 6 mice). E: average F1 score of binary decoder of behavior pairs trained on integration dimension activity (**p < 0.005, *p<0.01, n = 6 mice). F: cumulative distribution of integration dimension value (normalized) for different behaviors. G: projection of fastest dimension in example VMHvl mouse 1. H: performance of binary decoder of behavior pairs trained on fastest dimension activity (n = 6 mice). For additional data see Supplemental Figure S1, S2 and S3.

rSLDS first reduces neural activity to a set of latent variables (also called “dimensions” or “factors”), defining a low-dimensional “state-space” in which the time-evolving population neural activity vector can be analyzed (Figure 2A➀). Population activity in this low-dimensional space is then segmented into a set of discrete states (Figure 2A➁), while fitting a linear dynamical system model (Figure 2A➂) to neural activity within each state. Each state has a different dynamics matrix, which dictates how neural activity evolves over time from any given point within that state space. Quantitative examination of parameters from this matrix after model fitting can unveil dynamical properties of the neural circuit, such as the time constant of each dimension^42^. Finally, to visualize more easily the dynamical properties of each state, we plotted its “flow field” in 2D using principal component analysis (PCA) (Figure 2A➃ right, see Methods).

In fitting the rSLDS model, we chose the minimum number of states and dimensions that could capture 90% of observed variance in neural activity, determined using cross validation in each mouse separately (Supplemental Figure S1B–E; 7–8 dimensions (7.2± 0.1, N=6 mice) and 3–4 states). We evaluated the “goodness of fit” of each model iteration using both the log likelihood of the data^48^ and an additional metric that we call the “forward simulation error” (Supplemental Figure S1F, FSE, see Methods). Plotting the FSE over time allows visualization of periods wherein model performance drops (Supplemental Figure S1G). By this metric, our best-fit models captured most of the variance in neural data (model performance (1 – FSE) = 0.72 ± 0.02, N = 6 mice; Supplemental Figure S1H).

The rSLDS framework allows the fit dynamical system models to be either autonomous or to receive external input. Since VMHvl neuron firing rates correlate with the distance to another male or to male mouse urine^49^, likely reflecting the concentration of chemosensory cues^50^, we used the distance between animals and their facing angle as a proxy for external sensory input strength^49,51^ (see Methods).

### rSLDS analysis of VMHvl neural activity discovers an integration dimension that correlates with aggressive escalation

Next, we performed retrospective alignment of the unsupervised neural data model with behavioral annotations over time. This comparison revealed that the probability of attack was elevated during a single rSLDS state (state 3, Supplementary Figure S1I–K). Importantly, attacks were not time-locked to the onset/offset of this state; rather epochs of this state outlasted individual attack bouts (state 3 epoch duration: 79.5± 5.5s, attack bout duration: 4.86± 0.44s, N = 6 mice, Supplemental Figure S1I_5_, J_3_. K_3_). This suggests that the state did not simply represent motor activity (Supplemental Figure S1A, cf. Case 2 vs 1).

To understand better the neural population dynamics related to attack behavior, we examined the dynamics matrix for this state, which describes how dimensionally reduced neural activity in that state changes over time. The eigenvalues of this matrix reflect the rate at which activity along each of these dimensions decays to zero following external input, and can be converted to a time constant for each dimension^52,53^. Input to dimensions with short time constants will quickly decay to zero, whereas input to dimensions with long (large) time constants persists and decays slowly. Strikingly, one of the rSLDS dimensions had an estimated time constant of over 100 seconds that was significantly higher than that of all other dimensions (Figure 2B, red dot, 2C, 2D, N =6 mice). Because systems with long time constants approximately integrate their input over time, we refer to the longest time constant dimension as the “integration” dimension^54,55^.

The integration dimension accounted for 19.5%± 1.9% of the overall variance in neural activity (N = 6 mice). In contrast a support vector machine (SVM) decoder trained to distinguish attack from sniffing periods explained much less variance (0.3% ± 0.1%, N = 6 mice, p<0.001, Supplemental Figure S2B) Examining the activity of individual neurons that were weighted strongly in the integration dimension (Supplemental Figure S2D) revealed that around 20% of neurons per animal contributed to this dimension, with some showing ramping and persistent activity (Supplemental Figure S2I–J, L, N). Moreover most of these neurons were tuned to male intruders (Supplemental Figure S3A, B). Thus, the integration dimension encapsulates a signal that is present at the level of at least some individual neurons, but is also an emergent property of the population^47^.

We next compared the time-varying activity of the integration dimension with the animals’ actions during aggressive encounters. In mouse 1, activity along the integration dimension was low during sniffing, ramped up at the onset of dominance mounting (a low-intensity aggressive behavior^31^), and increased further to a stable plateau value as the animal attacked (Figure 2C). A cumulative distribution function (cdf) of the normalized level of activation along the integration dimension during sniffing, dominance mounting, and attack revealed that these three behaviors occurred at low, medium and high values of this dimension, respectively (Figure 2F; distribution means: sniffing: 0.30, dominance mount: 0.66, attack: 0.82, N = 6 mice).

Remarkably, a binary classifier created by thresholding the value of the integration dimension could distinguish periods of sniffing from attack, or from dominance mounting, with a high F1 score (0.89 ± 0.02, N=6 mice, Figure 2E). The same method could also distinguish dominance mounting vs. attack (F1 score 0.74 ± 0.03, N=6 mice, Figure 2E). However such classifiers could not distinguish behaviors occurring close together in time, such as attack and sniff-attack (defined as periods of sniffing that occurred within one second prior to attack, as described recently^56^), perhaps due to the gradual ramping of activity along this dimension. Remarkably, none of the other seven fit dimensions could be used to distinguish aggressive behaviors from sniffing with above chance accuracy (Figure 2G, H; Supplemental Figure S2C).

The foregoing analysis suggested that a low-dimensional signal in VMHvl represents escalating aggressive behaviors. To account for possible spurious behavioral correlations due to the slow decay of activity in this dimension, we devised a version of session permutation as described recently^57^, by cross validating decoder thresholds between animals (see Methods). This more rigorous paradigm could still decode behaviors with high F1 scores (Supplemental Figure S2H).

Sniffing, attack and dominance mounting are performed in bouts separated by short inter-bout intervals (IBIs). Because of its slow ramping and stable plateau, activity in the integration dimension did not decay during such IBIs and therefore could not distinguish behavioral bouts from adjacent IBIs (Supplemental Figure S1A, Case 1 vs 2). However decoders trained on this activity could distinguish IBIs from sniffing versus attack epochs, which were behaviorally indistinguishable to a human observer, with a high F1 score (0.83 ± 0.02, N=6 mice; Figure 2E, Supplemental Figure S1A, right, Case 2).

Thus, our unsupervised approach uncovered a one-dimensional signal in VMHvl^Esr1^ neural population activity that closely tracks and scales with an animal’s escalating level of aggressiveness and is reflected in the activity of approximately 20% of individual VMHvl^Esr1^ neurons. Different aggressive actions are observed as activity along this dimension reaches different thresholds, suggesting an aggression-intensity code in VMHvl^Esr1^ activity. The level of activity along the integration dimension could not be fully predicted from pose features such as the acceleration, facing angle, or velocity of the resident, or from the distance between mice (mean R^2^: 0.28 ± 0.04, N= 6 mice, Supplemental Figure S2A). Tracking metric used as inputs to the model were also not predictive of behavior annotations (Supplemental Figure S3F–H). Furthermore, models of VMHvl fit without any tracking inputs also recovered an integration dimension with similar time constants (Supplemental Figure S3D). These results further highlight that the relationship between the integration dimension and escalating aggressive behavior is not due to the incorporation of inputs such as facing angle and distance between mice. Even the incorporation of additional tracking metrics such as speed and area of the ellipse fit to the resident mouse did not improve rSLDS fits, suggesting that VMHvl was likely not integrating features of these sensory related signals (Supplemental Figure 3I).

This relationship between VMHvl^Esr1^ activity and aggression is consistent with our observation that increasing the intensity of optogenetic stimulation of VMHvl^Esr1^ neurons progressively evokes sniffing, dominance mounting and attack^12^, actions that can be decoded from the integration dimension as its activity ramps up.

### VMHvl contains an approximate line attractor that represents escalating aggressiveness

**Figure 3:**
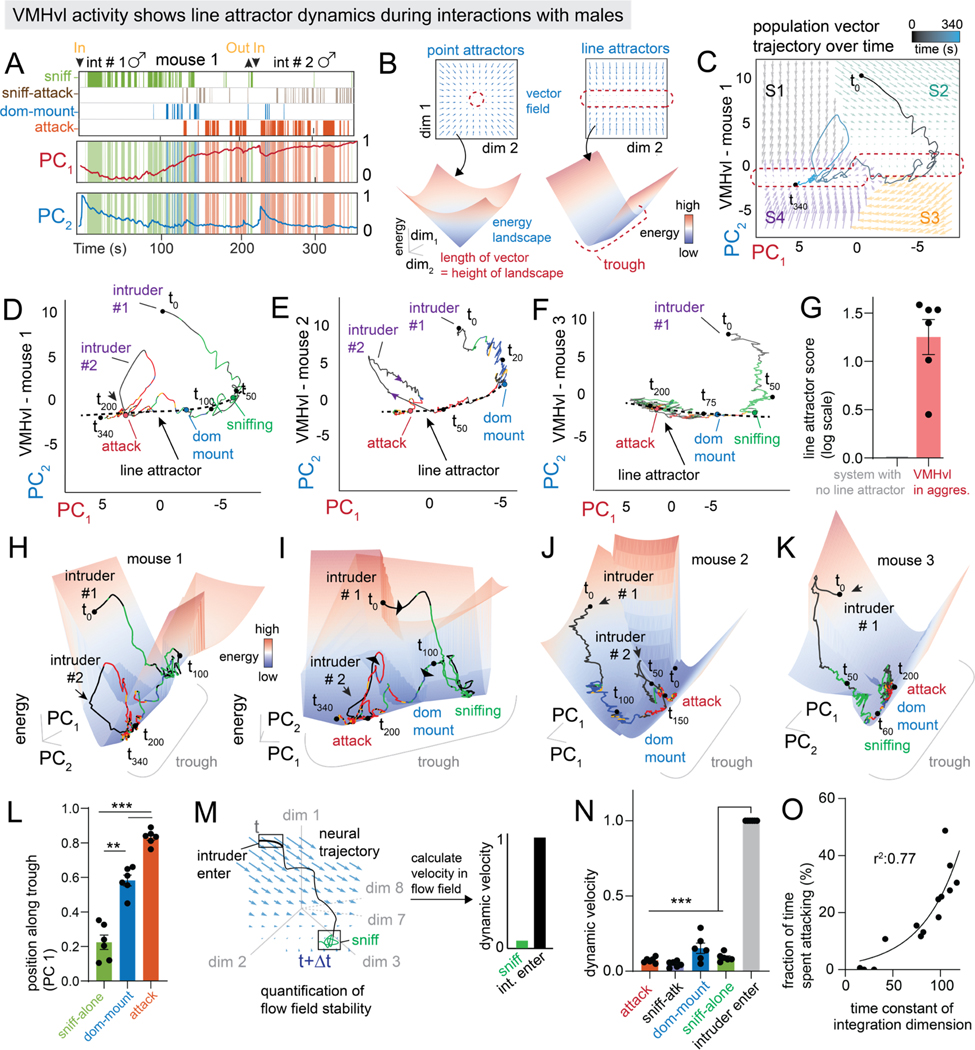
VMHvl contains an approximate line attractor that integrates aggressive escalation A: behavior rasters shown with first two principal components of dynamical system (see Methods) for example VMHvl mouse 1. B: inferred dynamics shown as a flow field (with attractor highlighted) and 3D landscape for point attractors (left) and line attractors (right). C: neural state space with population trajectories and inferred flow field colored by rSLDS states for VMHvl mouse 1, with line attractor highlighted. D-F: neural state space for VMHvl mouse 1 (D), mouse 2 (E) and mouse 3 (F) with line attractor highlighted (see Methods). G: line attractor score (see Methods) for VMHvl (red bar, n = 6 mice). H,I: inferred 3D dynamic landscape in VMHvl mouse 1(H,I). J,K: Same as H but for VMHvl mouse 2 (J) and mouse 3 (K) L: position of various behaviors along trough, i.e PC1 in neural state space (n = 6 mice, **p<0.005, *p<0.01) M: schematic showing quantification of dynamic velocity. N: dynamic velocity for various behaviors in VMHvl (***p<0.001, n = 6 mice) O: relationship between the time spent attacking and the time constant of the integration dimension of individual mice (r^2^: 0.77, n = 14 animals). For additional data see Supplemental Figure S4.

We examined next how the integration dimension of the fit model influences the overall topology of neural state-space during social behavior (Figure 3A, see Methods). PCA indicated that the first two PCs accounted for 68.5%± 1.2% of the total variance in VMHvl activity (N=6 mice). In all imaged animals, PC1 showed slow ramping dynamics (Figure 3A, Supplemental Figure S4C, PC_1_ (behavior-triggered average, N = 6 mice). We confirmed that the rSLDS integration dimension makes the largest contribution to this PC (Supplemental Figure S4A). Activity along PC2 was high when a new intruder was introduced (Figure 3A, Supplemental Figure S4C, PC_2_ (behavior-triggered average, N = 6 mice)), but was otherwise low.

To visualize neural state space dynamics, we next generated a 2D flow field in PC space, whose vectors at each point indicate how neural dynamics evolve according to the fit rSLDS model (see Figure 2A4). This revealed a region of low vector flow that forms an approximate line attractor (Figure 3B, *right*, 3C), meaning that the neural population activity vector tends to move towards persistent points along a line^58^ (Figure 3D, t_50_-t_340_).). To quantitatively delimit this attractor, we calculated the points in the flow field where vector length is at a minimum (“slow points;” see Methods) and linked these points into a dashed line (Figure 3D, dashed black line). Such approximate line attractors were observed in multiple mice (Figure 3E, F and Supplemental Figure 4D, E). Importantly, these line attractors are largely aligned with the PC1 axis, which principally reflects variance in the slow integration dimension identified by rSLDS (Figure 2B, Supplemental Figure S2A).

To quantitatively test for the existence of a line attractor in each mouse, we devised a “Line Attractor Score” as the base-2 log of the ratio of the largest to the second-largest time constants of the eight rSLDS dimensions (Figure 2C). According to basic concepts in dynamical systems theory^52^, this ratio has a relatively high value in systems containing a single integration dimension (forming an approximate line attractor), and is otherwise close to zero. We find that all mice with VMHvl recordings possess a line attractor score greater than zero, indicating the presence of a line attractor (Fig 3G, n = 6 mice).

As population activity progressed along the line attractor from low to high values of PC1, behavior progressed from sniffing to dominance mounting to attack (Figure 3D–F, 3L and Supplementary Video 1). This reflects the “ramping up” of activity seen in the integration dimension as social behavior progresses through these phases (Figure 2B), suggesting an encoding of an underlying continuous variable, as seen in line attractors in other regions^42,46,59–61.^

To visualize the dynamical topology of the rSLDS model, we represented the 2D flow field as a 3D landscape, by converting the length of the flow-field vectors at each position in neural state space into the height (z-axis) of the landscape (Figure 3B); the x-y axes are still represented by PC1 and PC2. In this topographic representation, a line attractor appears as a region shaped like a trough or gully, reflecting a slow rate of change (short vectors). A point attractor would appear as a locus of slow rate of change at the base of a cone (Figure 3B^59^). We observed a trough-like structure in the 3D dynamics landscape in each imaged animal (Figure 3H–K, Supplemental Figure S4P), along which neural activity progressed slowly as aggression escalated (Supplementary Video 2). Consistent with the persistent, slow-decaying activity characteristic of “leaky” neural integrators^55,59^, VMHvl activity remained high following intruder removal, and slowly decayed along the trough of the attractor over tens of seconds (Supplemental Figure S4H–J).

Although the animals’ behavior appears to occur while the system is in the line attractor, it could be that other rSLDS dimensions also show a change in their activity during behavior. To test this possibility, we computed each behavior’s “dynamic velocity”, by calculating the average vector length across all eight rSLDS dimensions at all time points in which a given behavior occurred. (Figure 3M, see Methods). Time points associated with initial intruder entry had the highest dynamic velocity and were present on the walls of the trough (Figure 3H–K), whereas aggressive behaviors exhibited low dynamic velocities and were distributed along the base of the trough (Figure 3N).

Once the system is in the line attractor, input that is not aligned with the attractor should produce a transient excursion of the population activity vector out of the trough; however once that input decays the vector should move back into the trough close to where it started from^42^ (Supplemental Figure S4F). We tested this prediction using a subset of experiments in which one intruder male was removed, and a second male introduced 30–60 seconds later. Strikingly, the introduction of a new intruder male drove a rapid rise in neural firing rates that pushed VMHvl activity away from the trough of the line attractor (Figure 3C–E, intruder #2). However, this signal decayed relatively quickly and the system re-entered the line attractor at nearly the same point (Supplemental Figure S4F–J and Supplemental Video 1). Importantly, the system recovered to the point in the attractor where it had been prior to introduction of the second intruder, regardless of when in the trial the first intruder was removed (Supplemental Figure S4K).

### The time constant of the integration dimension in VMHvl predicts levels of aggressiveness across animals

Although VMHvl^Esr1^ imaging data from different mice always revealed a single integration dimension with a long time constant, the magnitude of this time constant varied across individuals. Unexpectedly, we observed a trend in which animals that displayed more aggressive behavior (calculated as the fraction of time spent attacking) also exhibited an integration dimension with a longer time constant (Figure 3O, r ^2^ = 0.77, n = 14 animals). This relationship held for imaging data from different studies^29,31,39^ using different versions of GCaMP (6s vs. 7f; Supplemental Figure S4L–O). This striking correlation of integration time constant with time spent attacking suggests that individual differences in aggressiveness may be reflected in the intrinsic dynamics of VMHvl^Esr1^ neurons.

### Mating behaviors are represented using rotational dynamics in the MPOA

Since rSLDS was able to uncover evidence for integration in VMHvl, we next examined whether the same analysis would uncover population dynamics important for mating in MPOA, by fitting models to MPOA^Esr1^ neural data recording during interactions with female intruders (Karigo et al. 2021).

**Figure 4:**
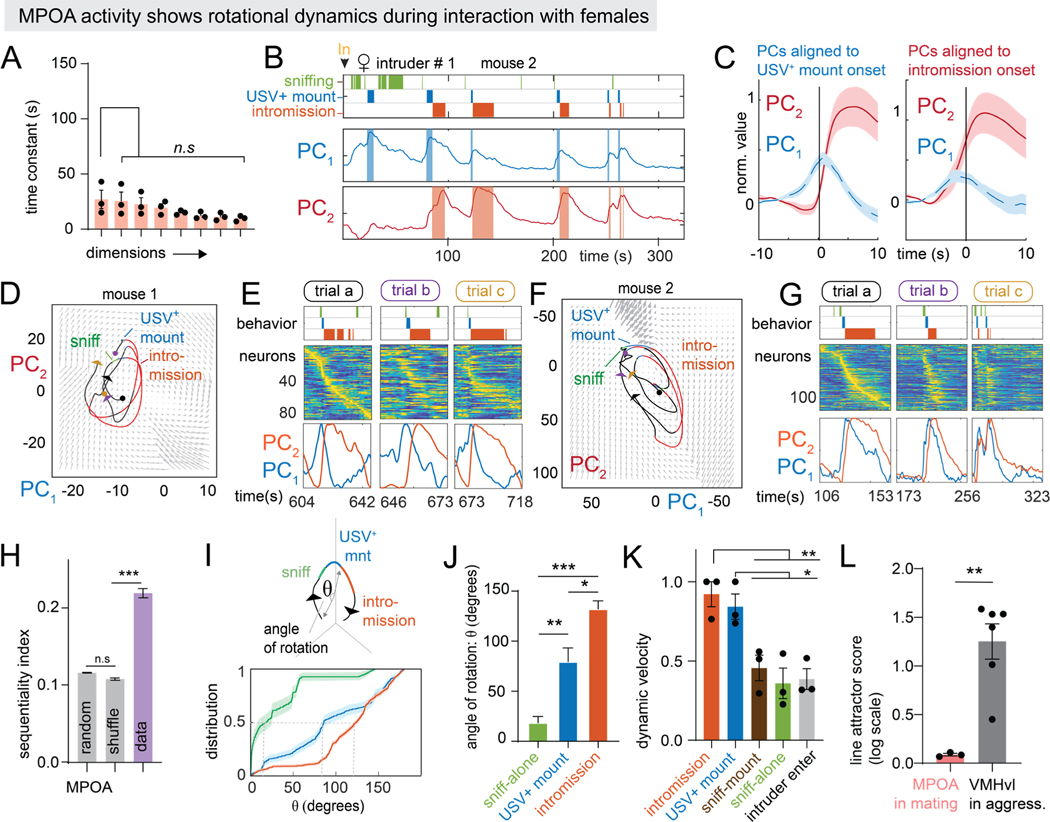
Mating behaviors are represented using rotational dynamics in the MPOA A: time constants of rSLDS dimensions in mating behavior-enriched state in MPOA (n = 3 mice) B: behavior rasters shown with first two principal components of latent factors for example MPOA mouse 2. C: behavior triggered average of top two principal components aligned to USV+ mount onset (left) and intromission (right) onset (n = 3 mice). D: neural state space with rotational population trajectories from mating episodes shown in E of MPOA mouse 1, colored by behaviors performed by resident mouse. E: sequential activity of MPOA neurons during mating episodes whose rotational population trajectories are shown in D. F,G: same as D,E but for MPOA mouse 2. H: sequential index for MPOA (n = 3 mice, ***p<0.001). I: calculation of angle of rotation (θ) aligned to the start of sniffing during mating episodes (top). Empirical cumulative distribution of θ for various behaviors (n = mice, bottom). J: quantification of θ for various mating behaviors (n = 3 mice, ***p<0.001, **p<0.005, *p<0.01, top). Schematic depicting θ for mating behaviors (bottom). K: dynamic velocity for mating behavior in MPOA (n = 3 mice).. L: line attractor score for MPOA activity in mating behaviors towards females (left, pink bar, n = 3 mice) and VMHvl activity in aggressive behavior towards males (right, grey bar, n = 6 mice, **p<0.005, data from Fig 3G reproduced for comparative purposes). For additional data see Supplemental Figure S5.

Fit models of MPOA required three rSLDS states in every animal, with mounting and intromission mostly occurring in single but different states (Supplemental Figure S5A–J). Unlike in VMHvl, the bout length of mating behaviors was similar to that of the corresponding state (Supplementary Figure S5 D, E). Strikingly, the eigenvalues of the dynamics matrix for such states did not include dimensions with long time-constants (Figure 4A). Instead, the first two PCs of the fit model revealed fast dynamics that were highly correlated with specific behaviors (Figure 4B). PC1 peaked at the onset of USV^+^ mounting bouts, while PC2 peaked during intromission (Figure 4B, C, behavior triggered average, N = 3 mice).

The 2D flow-field in PCA space revealed that neural dynamics were dominated by a rotational flow, with activity during mating epochs exhibiting periodic orbits (Figure 4D, F). The phase of the rotations was correlated with progression through sniffing, mounting, and intromission (Figure 4D, F, Supplemental Figure S5K–M), and corresponded to the sequential activation of different neurons during these successive behaviors (Figure 4E, G, Supplemental Figure S5A). Accordingly, the “sequentiality index” of the data^62^ was significantly greater than shuffled data or random matrices of similar sizes (seq. index = 0.22 ± 0.01, N = 3 mice, shuffle seq. index = 0.10± 0.002, N = 3 mice, Figure 4H).

We assessed the relationship between the phase of rotational trajectories and behavior by calculating the angle of the population activity vector relative to its value at the start of sniffing (Figure 4I). This revealed that sniffing, mounting and intromission occurred at characteristic angles of the population vector (sniffing: 18.6° ± 6.2 °, mounting: 79.61°±13.6 °, intromission: 132.2°± 8.1 °, N = 3 mice; Figure 4J). High dynamic velocities were associated with mounting and intromission, in striking contrast to the low dynamic velocity during attack behavior in VMHvl (Figure 3N, 4K).

To quantitatively assess the presence of line attractor dynamics, we computed the Line Attractor Score for MPOA. These values were close to zero and significantly different from those for VMHvl during aggression (Figure 4L). Thus, unlike the slow ramping and persistent dynamics identified in VMHvl, rSLDS discovered fast, sequential and behaviorally time-locked rotational dynamics in MPOA.

**Figure 5:**
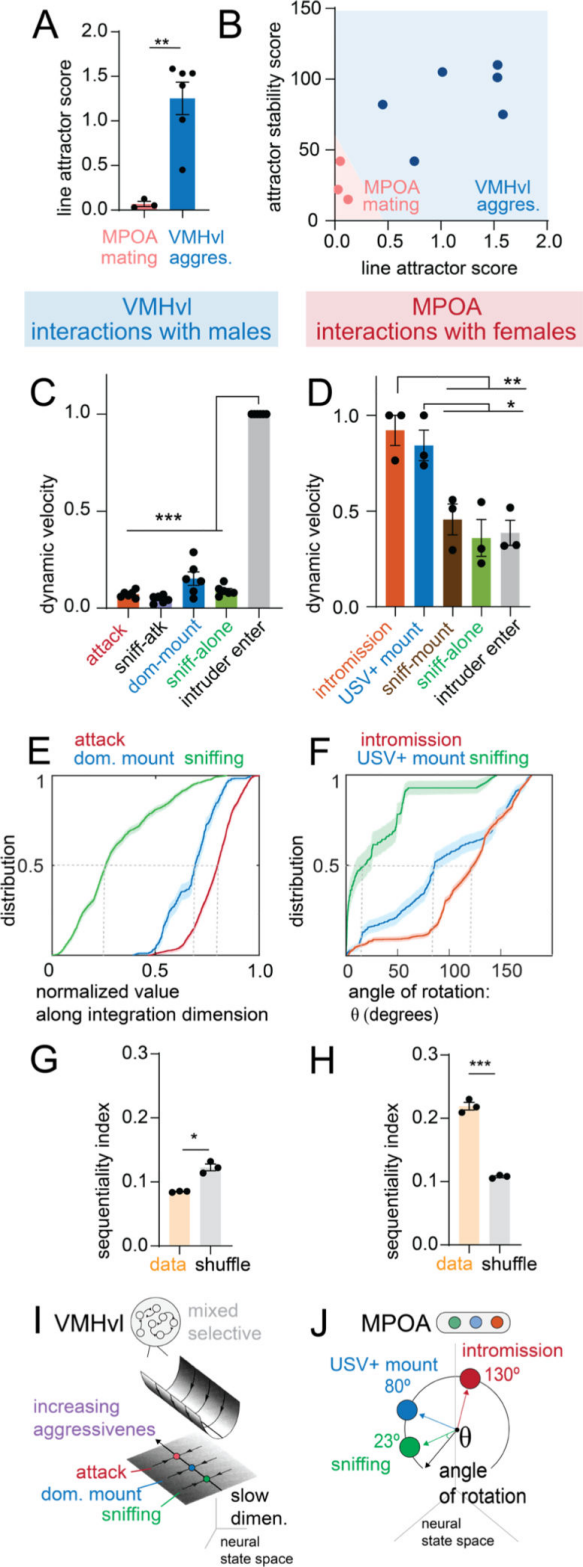
Distinct neural coding schemes for similar behavior in VMHvl vs MPOA A: line attractor score for mating behavior in MPOA and aggressive behavior in VMHvl (n = 3 mice for MPOA, n = 6 mice for VMHvl), reproduced from Figure 4L. B: scatter plot for line attractor score versus attractor stability score (magnitude of largest time constant) separates VMHvl and MPOA. C,D: dynamic velocity score in VMHvl during aggression (C) and MPOA during mating (D), reproduced from Figure 3N and Figure 4K respectively. E: empirical cumulative distribution of value of integration dimension (normalized) in VMHvl for various aggressive behaviors, reproduced from Figure 2F. F: empirical cumulative distribution of angle of rotation (normalized) in MPOA for various mating behaviors, reproduced from Figure 4I. G,H: Sequentiality index in MPOA (n = 3 mice), reproduced from Figure 4E, and in VMHvl (H) in aggression (n = 3 mice). I: summary of line attractor dynamics in VMHvl. J: summary of rotational dynamics in MPOA.

A direct comparison of key quantitative dynamics parameters highlights the key differences between VMHvl and MPOA (Figure 5A–D, G-H). Nevertheless in both regions evolving behavior tracks a single continuous variable: the value of the integration dimension in VMHvl, and the angle of the orbit in MPOA (Figure 5E, F). These variables are instantiated as a line attractor vs. rotational flow, respectively (Figure 5I, J).

### VMHvl exhibits an approximate line attractor encoding reproductive behavior

The foregoing findings raised the question of whether the contrasting dynamics in VMHvl vs. MPOA reflect differences specific to aggression vs. mating, or rather generic differences in behavioral coding between these nuclei. To address this, we fit rSLDS models to VMHvl^Esr1^ and MPOA^Esr1^ neuronal activity during mating vs. aggression, respectively.

**Figure 6:**
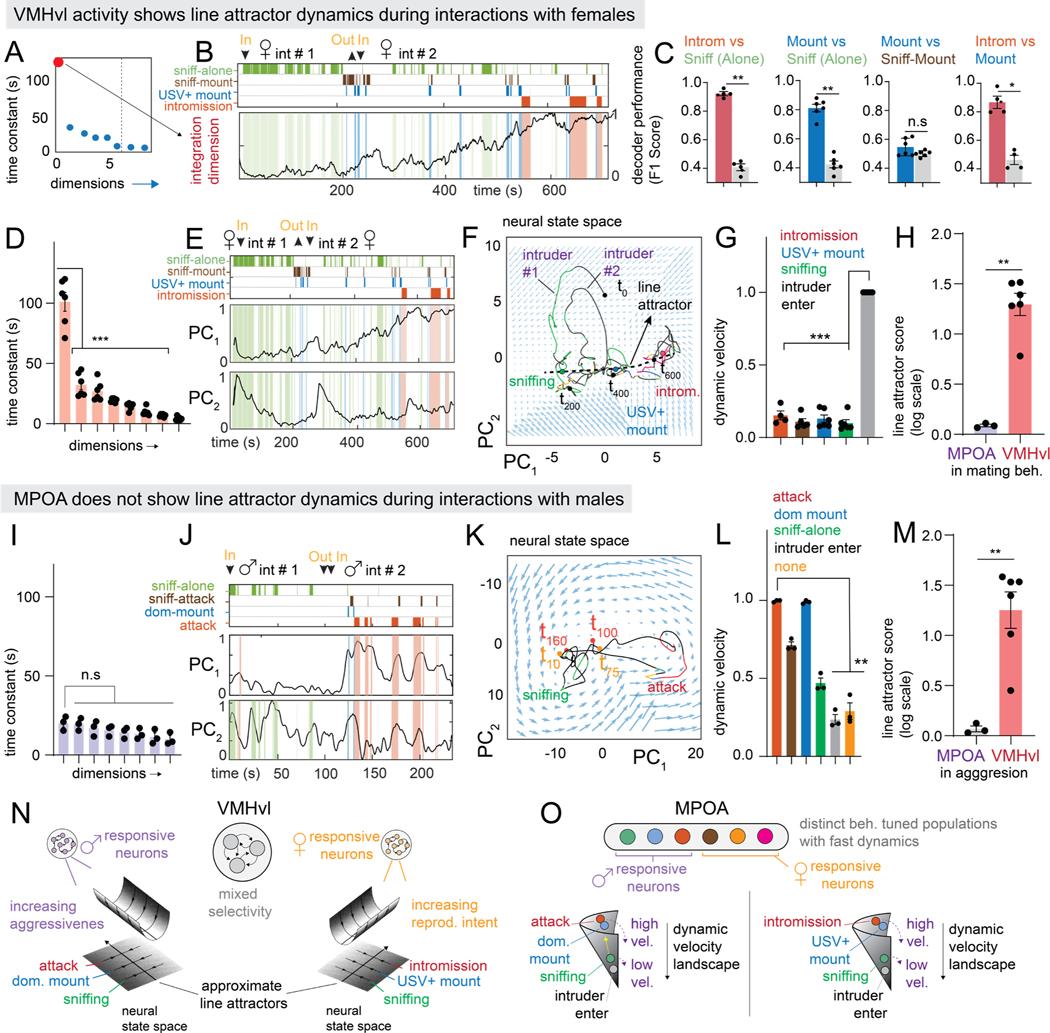
Distinct coding schemes of VMHvl and MPOA are region-specific, not intruder-specific A: left: time constants of rSLDS dimensions of mating enriched state from example VMHvl mouse 1. The red dot highlights the integration dimension. B: projection of integration dimension with overlayed behavior annotations. C: F1 score for decoding behavior pairs from integration dimension (**p < 0.005, *p<0.01, n = 4 mice for comparisons involving intromission as only 4/6 mice showed this behavior. n = 6 mice for all other comparisons). D: time constant arranged in decreasing order. (p < 0.001, n = 6 mice). E: behavior rasters shown with PCs of dynamical system for example VMHvl mouse 1. F: neural state space with population trajectories for VMHvl mouse 1 colored by behavior annotations and flow field showing a line attractor. G: quantification of dynamic velocity during mating behavior in VMHvl (p<0.001, n = 6 mice). H: line attractor score for MPO (n = 3 mice) and VMHvl (n = 6 mice) during mating behavior with females (**p<0.005). I: time constants of rSLDS dimensions from MPOA during aggression. J: behavior rasters shown with PCs of dynamical system for example MPOA mouse 1. K: neural state space with population trajectories for MPOA mouse 1 colored by behavior annotations and flow field. L: dynamic velocity during aggressive behavior in MPOA (**p<0.005, n = 3 mice). M: line attractor score for MPO (n = 3 mice) and VMHvl (n = 6 mice, reproduced from Fig 3G) during aggressive behavior (**p<0.005). N: Schematic illustrating two line attractors discovered in VMHvl encoding aggressiveness and mating intent. 0: Schematic illustrating dynamics seen in MPOA showing similarity in stability of behaviors during interactions with males and females. For additional data see Supplemental Figure S5.

Models fit to VMHvl activity during male-female encounters yielded a single integration dimension with a long time constant, created by neurons that displayed ramping and persistent activity (Figure 6A red dot, 6B, 6D, Supplemental Figure S6L). In addition, the duration of the rSLDS-discovered mating states in VMHvl tended to outlast individual bouts of mating actions (Supplemental Figure S6D, H), similar to the case of aggression in VMHvl (Supplementary Fig. 1I–K).

The cumulative distribution of the value of the integration dimension during various behaviors revealed that sniffing occurred at the lowest values, USV^+^ mounting at intermediate values, and intromission at the highest values of this dimension (Supplemental Figure S6J). Strikingly, pairwise decoders trained on this dimension performed with high accuracy (intromission vs sniffing: F1 = 0.92± 0.01 N = 4 animals; mounting vs sniffing: F1=0.81± 0.02 N = 6 animals Figure 6C). Such decoders could also distinguish periods of non-interaction between mounting bouts from those between sniffing bouts (Supplementary Figure S6I). Thus, VMHvl^Esr1^ neuronal dynamics during mating resembled those exhibited during aggression. However, the integration dimension seen during mating was biased towards neurons tuned to female intruders^29^, while male-tuned neurons primarily contributed to this dimension during aggression (7.73% 土 0.8% overlap, n = 6 mice, Supplemental Figure S6M, Supplemental Figure S3A,B).

As for aggression, a single dimension of the rSLDS model for mating exhibited a long time constant, yielding a high Line Attractor Score (Figure 6D, H). The first two PCs of the fit model were similar to those seen during aggression, with PC1 exhibiting ramping during the progression from sniffing to mounting to intromission (Figure 6E). Examination of the underlying 2D vector flow field revealed an approximate line attractor (Figure 6F) and a corresponding trough shape in the 3D dynamic velocity landscape, with neural activity moving along the trough as the animal progressed from the appetitive to consummatory phases of mating (Supplemental Figure 6K). Transient movements out of the line attractor occurred only during the introduction of a new intruder and were aligned with PC2 (Figure 6F, intruder #2). Accordingly, periods during intruder entrance had high dynamic velocities, while mating behaviors had low dynamic velocities (Figure 6G).

Thus rSLDS modeling of VMHvl^Esr1^ neuronal activity during mating revealed an approximate line attractor, with many features similar to those observed during aggression. However, the mating and aggression line attractors incorporate primarily female- vs. male-selective neurons, respectively (Supplemental Figure 6M). These data suggest that line-attractor dynamics are a general feature of social behavior coding in VMHvl, rather than a unique signature of aggression *per se*.

### MPOA does not exhibit line attractor dynamics during aggression

Finally, we fit rSLDS models to MPOA^Esr1^ neuronal dynamics during male-male encounters. Analogous to the case of mating behaviors, we found a state (state 3) that is closely aligned to the onset and offset of attack behavior (Supplementary Figure S6N). No dominant “slow” dimension was apparent in the time constants of the rSLDS dimensions (Figure 6I, M). Reflecting this, PC1 of rSLDS state space exhibited a fast increase in activity at the onset and offset of attack (Figure 6J, Supplemental Figure S6O, blue trace), in contrast to the slow attack-related dynamics in VMHvl (Supplemental Figure S6O, red trace).

Visualizing the 2D MPOA flow field in PC space revealed little change in the population trajectory during investigation (Figure 6K). During attack bouts, activity showed excursions into a separate region of state space, but quickly returned to the “sniffing” region after fighting (Figure 6K), reflecting the activation of different neuronal subsets (Figure 1E). Accordingly, attack and dominance-mounting had high dynamic velocities in MPOA, rather than the low dynamic velocities in VMHvl (Figure 6L, Supplemental Figure S6P). Lastly, the Line Attractor Score in MPOA during aggression had a value close to zero and was significantly different from that of VMHvl (Figure 6M, n = 3 for MPOA, n = 6 mice for VMHvl), confirming the absence of line attractor dynamics.

In MPOA, therefore, we find a representation of male-male encounters that alternates between investigatory and aggressive states, with the latter largely time-locked to the onset and offset of attack bouts. Strikingly, MPOA activity during aggression lacks the persistence, ramping and line attractor dynamics seen in VMHvl. Together with our analysis of VMHvl activity during mating, these results support the conclusion that MPOA and VMHvl exhibit fundamentally different coding of the same social behaviors.

## Discussion

### MPOA and VMHvl control social behaviors using different population codes

Here we report that MPOA^Esr1^ and VMHvl^Esr1^ neurons utilize very different schemes for the neural coding of mating and aggression, despite the fact that optogenetic perturbation specifically elicits mating in MPOA^Esr1^ neurons and attack in VMHvl^Esr1^ neurons. GCaMP imaging of Esr1^+^ neurons in MPOA indicates that specific actions can be decoded according to which cells are active^31^, consistent with transcriptomic studies^33^. In contrast, most VMHvl^Esr1^ neurons exhibit mixed behavioral selectivity in both imaging and transcriptomic studies^29,63^. Thus, MPOA represents behavior via a cell identity code, while VMHvl apparently does so via population coding.

Our studies suggest a possible mechanism underlying this population code. rSLDS analysis of VMHvl neural activity during male-male social interactions revealed one dimension of neural activity with a long time-constant that exhibits progressively increasing activity during escalating aggressive encounters. In a topological representation, these dynamics can be visualized as a progression along a stable “trough” or gully, which has the characteristics of an approximate line attractor^59^. In contrast, rSLDS analysis of MPOA revealed rotational dynamics, generated by the sequential activity of behavior-specific cell types during each bout of mating. Put simply, VMHvl coding of behavior appears to be analog, while MPOA coding of behavior appears more digital.

In other neural systems, line attractors often encode a continuous, low-dimensional variable^42,46^. Here, this variable may correspond to the intensity of an aggressive internal state. VMHvl neurons have previously been implicated in the motivation to engage in fighting, as operationalized using instrumental conditioning assays^23^. However such assays cannot measure aggressive motivation during attack itself, for technical reasons. The escalating (scalable) nature of aggression has ethological relevance as a means of establishing dominance while minimizing the risk of injury^64^. Unexpectedly, in comparing data across multiple animals we discovered a strong positive correlation between each mouse’s level of aggressiveness and the magnitude of the time constant of its integration dimension. This result reveals a neural correlate of individual differences in aggressiveness within VMHvl.

The different neural codes for social behavior we have uncovered in VMHvl and MPOA may reflect their distinct neurochemical and cytoarchitectonic features. VMH neurons are primarily glutamatergic. Recurrent connectivity among excitatory neurons is often invoked as a mechanism to achieve persistent activity^54,55,59^. Indeed, there is evidence that glutamatergic neurons in VMHdm that encode persistent defensive behaviors exhibit local connectivity^65^. However, slow dynamics can also be achieved using neuromodulatory signaling, and there is indirect evidence for peptidergic transmission in VMHvl^63,66^

By contrast, MPOA neurons are 85% GABAergic; to our knowledge there is no way to achieve similar graded and persistent signals within a population of inhibitory neurons. However, GABAergic neurons could provide a substrate for reciprocal inhibitory connections between action-specific subpopulations. Such connectivity could produce winner-take-all dynamics or feed-forward dis-inhibitory circuits that control transitions between sequential action phases of mating, e.g., from sniffing to mounting^39^, giving rise to the rotational dynamics observed in neural data. The existence of such circuits in MPOA can be investigated using slice physiology or in vivo imaging once genetic access to the appropriate cell types is achieved.

Why should MPOA and VMHvl utilize such different strategies for the coding of closely related social behaviors? It is tempting to attribute this difference in population dynamics to distinct features of reproductive vs aggressive behavior. For example, aggressive encounters can dynamically escalate or de-escalate to avoid serious injury or death to the combatants, whereas male mating must proceed to completion (ejaculation) to be reproductively beneficial. These differences are well-suited to control by ramping and rotational neuronal dynamics, respectively. In this view, the different properties and coding strategies of VMHvl and MPOA may have evolved to be optimally adaptive for fighting and mating, respectively.

However, our analysis also revealed approximate line attractor dynamics in a subset of VMHvl^Esr1^ neurons that is female-tuned and active during mating^29^. This suggests that line attractor-like dynamics are a general property of behavioral coding by VMHvl, not an aggression-specific feature. Conversely, MPOA contains specific Esr1^+^ neurons highly tuned to attack which do not exhibit line attractor dynamics (although there is no evidence that these neurons play a causative role in aggression). These data suggest that MPOA and VMHvl more likely encode different features of a given social behavior, such as action selection vs. motive state intensity, respectively. If so, then by extension the hypothalamus may contain GABAergic populations that control actionselection during aggression. Indeed, the anterior hypothalamic nucleus (AHN), which has a similar neurochemical and cytoarchitectonic structure as MPOA, can promote defensive attack^67,68^; it will be interesting to see whether rotational dynamics are observed in this structure. By the same token, PMv which controls aggression and is also primarily glutamatergic^50,69,70^, may utilize population coding like VMHvl.

### Potential functions of the VMHvl line attractor

Line attractors have been identified in cortical and hippocampal regions involved in cognitive functions, such as decision-making, spatial mapping and sensory discrimination^42,46^. It is unexpected to find such neural dynamics in the hypothalamus, which is widely viewed as controlling innate behaviors via action-specific cell types (as observed in MPOA^33^). What function(s) might such attractor dynamics serve, in the context of innate behaviors? Two explanations are possible, which are not mutually exclusive.

As mentioned earlier, progression along the line attractor may encode the intensity of an internal motive state of aggressiveness. This is supported by our finding that the integration dimension that contributes to this attractor can distinguish periods of nonsocial interaction during high- vs. low-intensity phases of aggressive escalation (Figure 2D). In this view, the line attractor serves to maintain the system in a stable internal motive state that persists continuously during stochastic expressions of observable attack.

Previous studies have indicated that the greatest source of variance in VMHvl^Esr1^ neural activity is intruder sex^29^. Whether VMHvl encodes intruder sex *per se*, or an internal motive state tightly correlated with intruder sex, has been difficult to distinguish because males only attack other males and not females. In female mice, however, lactating mothers attack intruders of both sexes. Recently, we identified a subset of VMHvl^Esr1^ neurons in females that express the GPCR gene *Npy2r*, called ß cells, which are both necessary for maternal aggression and sufficient to promote attack in non-aggressive virgins ^37^. Bulk calcium measurements revealed that ß cells are strongly active during maternal aggression towards both male and female intruders. However, these cells display low activity in individual females that are non-aggressive ^37^. Thus in females the encoding of aggressive state by VMHvl^Esr1^ neurons can be decoupled from the encoding of intruder sex. These data reinforce the idea that in males, the VMHvl^Esr1^ line attractor (which reflects a dimension weighted primarily by male-selective neurons) encodes aggressiveness, rather than simply intruder sex.

An alternative function for the line attractor is that it may serve as an integrator that accumulates “evidence” used to make behavioral decisions, such as the decision to switch from sniff to dominance mount, or from dominance mount to attack. Such a function would require that different behaviors be triggered at different threshold values of the integrator. This type of ramp-to-threshold mechanism has been suggested to control sequential actions during male courtship behavior in *Drosophila* ^71^ and predator escape in mice ^72^. These two hypotheses are not incompatible: the attractor could encode both the intensity of an internal state, and (indirectly) the selection of actions at different state intensities.

Line attractor dynamics could also serve useful functions in the context of behavioral plasticity and individual variation. For example, VMHvl^Esr1^ neurons show increased selective tuning for male vs. female intruders as a function of social experience ^29^, and exhibit a form of long-term potentiation that underlies the increase in aggressiveness that occurs when mice win a series of fights ^73^. It will be interesting to determine whether changes in flow field dynamics or attractor properties are associated with these forms of experience-dependent plasticity. Finally, we note that differences in line attractor properties were observed among mice which exhibited different and characteristic levels of aggressiveness (Figure 3O). It is possible that individual differences in aggressiveness may reflect, or be caused by, individual constraints on population dynamics in VMHvl.

### Testable predictions of the line-attractor model

Our rSLDS model of VMHvl dynamics makes several testable predictions and raises several interesting questions for future investigation. First, it predicts that once in the attractor, the system will return quickly to it following perturbations that move it out of this stable trough. This behavior is suggested by the brief excursion out of the attractor that occurs when a first intruder is removed and replaced by a second one. However, it would be ideal to demonstrate this directly by transiently activating neurons that contribute to the attractor, and determining whether the system rapidly returns to it following stimulus offset, as has been demonstrated for point attractors underlying working memory in ALM ^74^. Another prediction is that selectively inactivating the VMHvl^Esr1^ neurons that exhibit slow dynamics should eliminate activity along the line attractor. Such experiments will require combined optogenetic perturbations and calcium imaging in this deep subcortical structure. Such experiments will also be critical to confirm whether line attractor properties indeed play a causative role in controlling levels of aggressiveness.

The results herein show that about 20% of VMHvl^Esr1^ neurons exhibit persistent activity and ramping dynamics, raising the question of whether these cells constitute a genetically determined subpopulation. Single-cell RNAseq experiments have shown that the Esr1^+^ population in VMHvl can be subdivided into 6–7 distinct transcriptomic subtypes ^63^. Whether any of these subtypes selectively contributes to attractor dynamics can be addressed once genetic drivers specific for these subtypes are available. An additional question is whether the slow dynamics observed for some VMHvl^Esr1^ neurons reflects recurrent connectivity between them, as has been demonstrated for fear-encoding neurons in VMHdm ^65^, or the release of slow-acting neuromodulators such as neuropeptides. Recurrent connectivity in VMHvl can be investigated by slice electrophysiology^66^ and ultimately by EM connectomics. VMHvl^Esr1^ neurons are known to express multiple neuropeptides, as well as receptors for neuropeptides and other neuromodulators. New sensors for detecting neuromodulator release ^75,76^, as well as methods for dynamically perturbing neuromodulator function *in vivo*, should help to address these questions in the future.

### Limitations of the study

Our discovery of line attractor dynamics in VMHvl derives from quantitative analysis of a dynamical system model fit to neural data. While this analysis has revealed several conditions required for line attractor dynamics, such as persistence in the absence of input and robustness to behavioral perturbation, a definitive test requires experimental perturbation of neural activity^58^. Perturbations are also required to determine the contributions to line attractor dynamics of region-intrinsic vs extrinsic (i.e via other nuclei) recurrent dynamics and feedback, as well as whether the attractor is truly “autonomous” and not input-driven. The biological line attractor in VMHvl is a ‘leaky’ approximation of a mathematically defined line attractor, exhibiting slow decay over time scales similar to line attractors discovered in other neural systems^46,59^. Further knowledge of the underlying neural mechanisms is required to understand the extent to which the region of stability identified here approximates a true line attractor.

## STAR Methods

### Resource Availability

#### Lead Contact

Requests for resources and reagents should be addressed to lead contact, David J. Anderson (wuwei@caltech.edu).

#### Materials availability

This study did not generate new unique reagents.

#### Data and code availability

- Source data used in this paper will be shared by the lead contact upon request.
- Code used for analyses in this paper is available in the following repositories: https://github.com/lindermanlab/ssm https://github.com/DJALab/VMHvl_MPOA_dynamics
- Any additional information required to reanalyze the data reported in this paper is available from the lead contact upon request.

### Experimental model and subject details

#### Neural imaging data (Karigo et al., 2021, Remedios et al., 2017, Yang and Anderson, 2022)

We analyzed data from three sets of previous experiments ^29,31,39^ All experiments were approved by the Institute Animal Care and Use Committee (IACUC) and the Institute Biosafety Committee (IBC) at the California Institute of Technology (Caltech). All experiments utilized heterozygous *Esr1*^*cre/+*^ knock-in mice on a C457BL6/N background (B6N.129S6(Cg)-*Esr1*^*tm1.1(cre)And*^*I*J, JAX strain #017911). Expression of GCaMP6s (Remedios et al., 2017, Karigo et al., 2021) or GCaMP7f (Yang et al. 2022) was achieved by stereotaxic injection of a Cre-dependent GCaMP-expressing adeno-associated viruses (AAVs). Briefly, for data obtained from Karigo et al., 2021, mice expressing GCaMP6s selectively in *Esr1* neurons in either the medial preoptic area (MPOA) or the ventrolateral subdivision of the ventromedial hypothalamus (VMHvl), were allowed to interact with BALB/c male and female intruders in a standard resident intruder assay (Karigo et al., 2021). Male or female intruders were introduced into the home cage in a random order, with a 5–10 min interval between intruder session. Each session typically lasted 10–20 minutes. Behavior videos of interacting animals were annotated using a custom MATLAB-based interface. A total of 7 behaviors including sniffing, dominance-mount, attack, mount, intromission, interact (periods where animals were close to each other but other behaviors were absent) were annotated with male and female intruders. A head-mounted micro-endoscope (Inscopix, Inc.) was used to acquire Ca^2+^ imaging data at 15Hz from either MPOA^Esr1^ neurons (total of 583 neurons from 3 mice) or VMHvl^Esr1^ neurons (total of 421 neurons from 3 mice) for neural data analysis described in sections below.

For data obtained from Yang et al., 2022, Esr1-Cre mice in which GCaMP7f was expressed selectively in *Esr1* neurons in VMHvl, were allowed to interact with BALB/c male intruders in a standard resident intruder assay. In addition to the behaviors annotated for above, male intruders were also “dangled”, where the ano-genital region of the dangled intruder is held next to the resident mouse. A head-mounted micro-endoscope was used to acquire Ca^2+^ imaging data at 30Hz from VMHvl^Esr1^ neurons (386 neurons from 3 mice) for neural data analysis described in sections below.

For data obtained from Remedios et al, 2017, Esr1-Cre mice in which GCaMP6s was expressed selectively in *Esr1* neurons in VMHvl were allowed to interact with BALB/c male intruders in a standard resident intruder assay. A head-mounted micro-endoscope was used to acquire Ca^2+^ imaging data at 30Hz from VMHvl^Esr1^ neurons (358 neurons from 3 mice) for neural data analysis described in sections below. rSLDS models were fit to data from n=14 mice to extract the time constant of the integration dimension used for correlation with individual differences in aggressiveness in Figure 3O. However 8 of those mice were excluded from decoder analysis of sniffing, mounting and attack, either because they were highly aggressive and attacked without any prior sniffing or dominance mounting (5 mice), or because they were non-aggressive and failed to attack (3 mice). Typically 20–25% of male mice from the C57BL6 background fail to show aggression in resident-intruder assays (Stagkourakis et al., 2020).

### Method Details

#### Tuning rasters for single neurons

We examined the tuning properties of single neurons in VMHvl^Esr1^ or MPOA^Esr1^ by creating behavior tuning rasters (Figure 1 C, D). We first computed the mean activity of each neuron for each of the 14 manually annotated behavioral actions. To group neurons, we created a set of 40 regressors representing combinations of behavioral actions, and grouped neurons by which single regressor captured the most variance in each cell’s activity. In addition to regressors for individual behaviors, example regressors include signals such as all male-directed actions, all female-directed actions, all male-directed/female-directed/sex-invariant investigative behaviors, and all male-directed/female-directed/sex-invariant consummatory behaviors. Neurons for which no single regressor captured at least 50% of variance in behavior-averaged activity were omitted from the visualization (approximately 5% of cells.)

#### Computation of pose features for input to dynamical model

As external input to the dynamical model (see next section), we selected two features of animal pose estimates produced by the Mouse Action Recognition System (MARS, ^51^ The first of these is the distance between animals, computed as the distance between centroids of ellipses fit to the poses of the two mice. The second is the facing angle of the resident towards intruder mouse, defined as the angle between a vector connecting the centroids of the two mice and a vector from the centroid to the nose of the resident mouse. In addition we also fit dynamical models with either no input or with additional inputs in the form of the speed of the resident (computed as the mean change in position of centroids of the head and hips, computed across two consecutive frames) and area of ellipse fit to the resident mouse’s pose.

#### Dynamical system models of neural data

We model neural activity using a recurrent switching linear dynamical systems (rSLDS) according to previous methods^48,77^. Briefly, rSLDS is a generative model that breaks down non-linear time series data into sequences of linear dynamical modes. The model relates three sets of variables: a set of discrete states (z), a set of continuous latent factors (x) that captures the low-dimensional nature of neural activity, and the activity of recorded neurons (y). The model also allows for external inputs (u) which consists of extracted pose features including the distance between animals and the facing angle between the resident and intruder mouse.

The model is formulated as follows: At each time $\text{t}=1,2,\ldots {\text{T}}_{\text{n}}$, there is a discrete state $z_{t}\in \{1,2,\ldots ,K\}$.. In a standard SLDS, these states follow Markovian dynamics, however rSLDS allows for the transitions between states to depend recurrently on the continuous latent factors (x) and external inputs (u) as follows:

(1) $$p\left(z_{t+1}=k,z_{t}=j,x_{t}\right)\propto exp\left\{Rx_{t}+Wu_{t}+r\right\}$$

where *R*, *W* and *r* parameterizes a map from the previous discrete state, continuous state and external inputs using a softmax link function to a distribution over the next discrete states.

The discrete state *z*_*t*_ determines the linear dynamical system used to generate the continuous latent factors at any time t:

(2) $$x_{t}=A_{z_{t}}x_{t-1}+V_{z_{t}}u_{t}+b_{z_{t}}$$

where $A_{k}\in {\mathbb{R}}^{d\times d}$ is a dynamics matrix, $V_{Z_{t}}\in {\mathbb{R}}^{d\times m}$ is a matrix that describes the contribution of external inputs (𝑢_*t*_) to each dimension of the latent space and $b_{k}\in {\mathbb{R}}^{d}$ is a bias vector, where *d* is the dimensionality of the latent space and *m* is the dimensionality of the external inputs. Thus, the discrete state specifies a set of linear dynamical system parameters and specify which dynamics to use when updating the continuous latent factors.

Lastly, we can recover the activity of recorded neurons by modelling activity as a linear noisy Gaussian observation $y_{t}\in {\mathbb{R}}^{N}$ where N is the number of recorded neurons:

(3) $$y_{t}=Cx_{t}+d$$

For $C\in {\mathbb{R}}^{N\times D}$ and d∼N(0,S), a gaussian random variable. Overall, the system parameters that rSLDS needs to learn consists of the state transition dynamics, library of linear dynamical system matrices and neuron-specific emission parameters, which we write as:

$$\theta =\left\{A_{k},V_{k},b_{k},C,d,R,W,r\right\}$$

These parameters are estimated using maximum likelihood using approximate variational inference methods as described in detail in ^48,77^.

Model performance is reported as the *evidence lower bound (ELBO)* which is equivalent to the Kullback-Leibler divergence between the approximate and true posterior, KL(q(x,z;φ)∥p(x,z∣y;θ)) using 5-fold cross validation.

Since the ELBO is sensitive to the inclusion of regularizers and the amount of data used during fitting, we also provide an additional “forward simulation error (FSE)” model evaluation metric calculated as follows: given observed neural activity in state space at time *t*, we predict the trajectory of the population activity vector over an ensuing small time interval Δ*t* using the model, then compute the mean squared error (MSE) between that trajectory and the observed data at time *t+* Δ*t* (Supplemental Figure S1F). This MSE is computed across all dimensions of the latent space and repeated for all times *t*. This error metric is normalized to a 0–1 range in each animal across the whole recording to obtain a bounded measure of model performance (Supplemental Figure S1F). This metric is computed across cross-validation folds and can provide intuition about time segments where model performance drops

Code used to fit rSLDS on neural data is available in the SSM package: (https://github.com/lindermanlab/ssm)

Code to generate flow fields and energy landscapes from fit dynamical systems is available in (https://github.com/DJALab/VMHvl_MPOA_dynamics)

#### Estimation of time constants

We estimated the time constant of each mode of linear dynamical systems using eigenvalues λ*_a_* of the dynamics matrix of that system, derived by ^53^ as:

$$\tau_{a}=\left|\frac{1}{\log\left(\left|\lambda_{a}\right|\right)}\right|$$

#### Calculation of line attractor score

To provide a quantitative measure of the presence of line attractor dynamics, we devised a line attractor score defined as:

$$line\;attractor\;score\;=\log_{2}\frac{t_{n}}{t_{n-1}}$$

where *t*_*n*_ is the largest time constant of the dynamics matrix of a dynamical system and *t*_*n*−1_ is the second largest time constant. This measure would be zero in a system without line attractor dynamics due to the similar magnitudes of the first two largest time constants and would be greater than one for systems that possess a line attractor.

#### Decoding behavior from integration dimension

We trained a frame-wise decoder to discriminate pairs of behavior (such as sniffing vs attack) from the activity of the integration dimension on individual frames of a behavior (sampled at 15Hz) as described previously (Karigo et al., 2021). We first created ‘trials’ from bouts of social behavior by merging all bouts that were separated by less than five seconds. We then trained a linear support vector machine (SVM) to identify a decoding threshold that maximally separates the values of our normalized “integration dimension” signal on frames during which behavior A occurred from values on frames during which behavior B occurred, for the pair-wise behavioral comparison. ‘Shuffled’ decoder data was generated by setting the decoding threshold on the same “trial”, but with the behavior annotations randomly assigned to each behavior bout. We repeated shuffling 20 times for each intruder and each imaged mouse. We report performances of actual and shuffled 1D-threshold “decoders” as the average F1 score of the fit decoder, on data from all other “trials” for each mouse. For significance testing, the mean accuracy of the decoder trained on shuffled data was computed across mice, with shuffling repeated 1000 times for each mouse. Significance is determined by bootstrapping; we considered observed F1 scores significant if they fell above the 97.5th percentile of the distribution of chance F1 scores as done previously ^29^.

As a stringent test for spurious correlations due to the slow decay seen in the integration, we performed a variation of session permutation ^57^ as follows. Consider a neural signal that displays a slow ramp in activity, which can be used to decode attack from sniffing Supplemental Figure S2E). If this correlation was spurious and occurred due to slow drift in activity, that decoding threshold would perform poorly if used on the integration dimension from another mouse (Supplemental Figure S2F). On the other hand, that same threshold would produce a high F1 score if the correlation was not spurious as shown in Supplemental Figure S2G. To implement this paradigm, we used the decoding threshold obtained in a given mouse on the integration dimension from all other mice and averaged the final performance.

#### Low dimensional (PCA) representation of dynamical system

Since the latent states are invariant to linear transformations, it is possible to apply a suitable transformation to obtain an equivalent model using rSLDS. We use PCA for this transformation as it allows us to describe our high dimensional rSLDS latent space in a concise manner with few dimensions while capturing the overall dynamics. To perform this the following steps are applied:

1. Given latent factors: $x_{1},x_{2},\ldots ,x_{t}$ of the raw neural data 𝑦_*t*_
2. Compute a whitening transformation *W* such that *Wx* is the identity
3. Compute the transformed linear dynamical system $x_{t}^{\prime }=Wx_{t}$ with new emission matrix $C^{\prime }=CW^{-1}$.
4. Compute the singular value decomposition (SVD) of the new emission matrix $C^{\prime }=USV^{T}$. Let $P=SV^{T}$, such that $P^{-1}=\frac{V}{S}$
5. Compute the final transformed latent states (i.e principal components) $x_{t}^{\prime \prime }=P^{-1}x_{t}^{\prime }=P^{-1}Wx_{t}$

In this final transformation, since the singular values are ordered, the first two components of $x_{t}^{\prime \prime }$ accounts for the most variance in the raw neural data $y_{t}$. This method of applying PCA also accounts for the emission matrix C of the fit dynamical system.

#### Dynamic velocity as a measure of stability in a dynamical system and visualization as 3D landscape

We devised a metric termed the “dynamic velocity” to quantify the average intrinsically generated rate of change of the fit dynamical system during a given behavior of interest. We first calculated the average norm of $A_{z_{t}}x_{t}$ for every value of $x_{t}$ associated with a given behavior, for a given state *z*. We then averaged this value across states, giving a definition of $V_{b}=\frac{1}{n(Z)}\sum_{z\in Z}\left(\frac{1}{n\left(T_{b}\right)}\sum_{t\in T_{b}}\left\|A_{z_{t}}x_{t}\right\|\right)$, where Z is the set of states, *T*_*b*_ is the set of all timepoints during which behavior *b* occurred, ∥⋅∥ is the Euclidean norm, and n(·) is the number of elements in a set. Finally, to facilitate comparison across animals, we normalized this value to a 0–1 range, with respect to its maximum across behaviors in each animal. Low values of this measure close to zero indicate regions with high stability while large values indicate unstable regions of neural state space.

We also converted the flow-fields obtained from rSLDS into a 3D landscape for visualization by calculating the dynamic velocity at each point in neural state space and using it as the height of a 3D landscape.

#### Quantification and statistical analysis

Data were processed and analyzed using Python, MATLAB, and GraphPad (GraphPad PRISM 9). All data were analyzed using two-tailed non-parametric tests. Mann-Whitney test were used for binary paired samples. Friedman test was used for non-binary paired samples. Kolmogorov-Smirnov test was used for non-paired samples. Multiple comparisons were corrected with Dunn’s multiple comparisons correction. Not significant (NS), P > 0.01; *P < 0.01; **P < 0.005; ***P < 0.001; ****P < 0.0001.

## Supplementary Material

1 Supplementary Figure 1: Unsupervised discovery of aggression-enriched states in VMHvl
Related to Figure 2
A: types of neural states identified by rSLDS. B1, B2: behaviors; Q0, Q1: periods of quiescence between behavior bouts; S0,S1,S2: rSLDS states. Case 1: rSLDS states cannot distinguish behavior vs internal states. Case 2: rSLDS reflects internal state-encoding due to persistence during behavioral quiescence. B: optimization of number of rSLDS states in example VMHvl mouse 1. Model performance is measured as ELBO (see methods). C: same as B, but for dimensionality. D: variance explained by dimension chosen in C. E: convergence of model performance. F: creation of a bounded model performance metric (forward simulation error, FSE, see methods). G: FSE for VMHvl mouse 1 & 2. H: average model performance (FSE) before and after training (n = 6 mice,***p<0.001).: I_1_: rSLDS states in VMHvl mouse 1. I_2_: comparison of rSLDS states with behaviors. I_3_: behavioral composition of rSLDS states. State 3 possesses the highest amount of attack behavior across mice (see panel J, K). I_4_: probability of attack aligned to the onset of state 3 (n = 6 mice). I_5_: timescale of behavior bouts and discovered states epochs. I_6_: state transition diagram from empirical transition probabilities. J: Same as F_2_, F_3_, F_5_ but for VMHvl mouse 2. K: Same as F_2_, F_3_, F_5_ but for VMHvl mouse 3.

2 Supplementary Figure 2: Characterization of aggression-integration dimension
Related to Figure 2
A: variance explained by a generalized linear model trained to predict integration dimension from pose-features including distance between mice, facing angle, speed, acceleration, and velocity of resident mouse (mean: 0.28 ± 0.04 R^2^, n = 6 mice). B: fraction of overall variance explained by integration dimension (purple) compared to variance explained by decoder dimension trained to distinguish attack from sniff bouts (integration dimension mean: 19.5%± 1.9%, attack decoder mean: 0.3% ± 0.1%, n= 6 mice, ***p<0.001). C: decoding behaviors from non-integration dimensions (average across dimensions, n = 6 mice). D: absolute rSLDS weight on integration dimension of VMHvl mouse 1 (cell number on x-axis), sorted by choice probability values for male vs female intruder encounter. E-G: paradigm to account for spurious correlations: decoding threshold obtained using integration dimension of mouse 1 (E, purple line) is used on integration dimension from mouse 2 (F). Spurious correlations lead to low F1 scores (F) while true correlations retain high F1 scores (G). H: decoding behaviors using paradigm described above (**p < 0.005, n = 6 mice) I: normalized activity of neurons times rSLDS weight for cells with significant weights for integration dimension of VMHvl mouse 1. J: example cells from I. K: integration dimension in VMHvl mouse 2. L: same as I for VMHvl mouse 2. M,N: Same as K,L for VMHvl mouse 3.

3 Supplementary Figure 3: Characterization of aggression-integration dimension and dependence on tracking feature based external inputs.
Related to Figure 2
A: aggression integration dimension in female and male trials in VMHvl Mouse 1. B: mean projection of neural activity from female vs male trials onto the aggression integration dimension (n = 6 mice, **p<0.005). C: low dimensional dynamics and flow field from model with no behavioral inputs included with line attractor highlighted. D: time constants from the fit dynamical system (n = 6 mice). E: line attractor score for VMHvl models without input. F: tracking features used in rSLDS shown alongside discovered states and integration dimension in VMHvl mouse 1. G: performance of decoder used to separate attack frames from sniff-alone frames using the distance between mice and facing angle of the resident. H: scatter plot of distance between mice and facing angle of resident. I: model performance (1-FSE) for different types of external inputs (n = 6 mice); current inputs = distance between animals, facing angle of resident. (***p<0.001).

4 Supplementary Figure 4: Properties of line attractor dynamics in VMHvl.
Related to Figure 3
A,B: absolute PCA weights of PC1(A) and PC2(B) on dimensions of dynamical system sorted by decreasing time constant in VMHvl mouse 1. C: behavior triggered average of top two principal components aligned to introduction of first intruder or first attack onset (n = 6 mice). D,E: low dimensional dynamics and flow field showing line attractor dynamics for VMHvl mouse 2 and mouse 3 with line attractor highlighted. F: schematic showing how perturbations orthogonal to a line attractor do not alter the position of the system. G: integration dimension in VMHvl mouse 1 (reproduced from Fig 2B) with attack bout (1) and inter-trial interval (2) highlighted. H: neural state space with line attractor highlighted in VMHvl mouse 1, showing the persistence of activity during the inter-trial interval shown in G. The introduction of intruder #2 acts as an orthogonal perturbation and activity returns to the same point along the attractor. I,J: Same as G,H for VMHvl mouse 2. K: neural state space with line attractor highlighted in VMHvl mouse 4. The introduction of intruder #2 occurs earlier in the trial when the animal displays sniffing behavior but results in a similar perturbation as above. L: relationship between fraction of time spent attack vs time constant of integration for animals with GCaMP7f recordings (n= 8 mice). M: integration dimension in VMHvl mouse 5 (GCaMP 7f) shows the same persistence and slow decay of activity. N: same as M for VMHvl mouse 4 (GCaMP 7f). O: line attractor score for mice with GCaMP7f recordings (***p<0.001). P: dynamics landscape for VMHvl mouse 4 (GCaMP 7f) showing a trough shaped landscape.

5 Supplementary Figure 5: Mating enriched states and rotational dynamics in MPOA.
Related to Figure 4
A: rSLDS states in MPOA mouse 1. B: comparison of rSLDS states with behavior in MPOA mouse 1 for period from t = 600s to t = 700s. C: behavioral composition of rSLDS states. D: probability of intromission and USV+ mounting aligned to the onset of state 2 and state 3 (also see panel I, J, n = 3 mice). E: timescale of behavioral bouts and states epochs. F: Reproduced from Figure 4D but with state-specific inferred flow-field colors. G: state transition diagram from empirically calculated transition probabilities. H: state and behavior raster for MPOA mouse 1 for entire recording. I_1_: same as H for MPOA mouse 2, selected mating bouts highlighted. I_2_: behavioral composition of rSLDS states (bottom). I_3_: timescale of behavioral bouts and states epochs. J_1–3_: same as I_1–3_ for MPOA mouse 3, selected mating bouts highlighted. K: rotational trajectories for 3 mating episodes in MPOA mouse 1. L: same as K, for mating bouts highlighted in highlighted in I_1_ for MPOA mouse 2. M: same as K, for mating bouts highlighted in highlighted in J_1_ for MPOA mouse 3.

6 Supplementary Figure 6: Dynamical analysis of VMHvl activity in mating behavior and MPOA activity in aggression.
Related to Figure 6
A: rSLDS states in VMHvl mouse 1 during interactions with female intruders. B: comparison of rSLDS states with behaviors. C: behavioral composition of rSLDS states. State 3 possesses the highest amount of mating behavior across mice (see panel H). D: timescale of behavior bouts and state epochs. E: state transition diagram from empirical transition probabilities. F-H: Same as B-D for VMHvl mouse 2. This mouse did not achieve intromission. I: decoding behaviors from integration dimension (**p<0.005). ). J: empirical cumulative distribution of value of integration dimension (normalized) for various behaviors. K: dynamics velocity landscape showing a progression of mating behavior along the trough for VMHvl mouse 1. L: normalized activity times rSLDS weight for cells contributing significantly to integration dimension of VMHvl mouse 1. M: absolute rSLDS weight on integration dimension of VMHvl mouse 1 during mating behavior (top, yellow dots) and aggression (bottom, black dots) sorted by choice probability values for male vs female intruder encounter. N: top: state and behavior raster for MPOA mouse 1 during aggressive behavior. State 3 is aligned closely to the onset of attack bouts, bottom: behavioral composition of discovered states. O: behavior triggered average of principal component 1 in VMHvl (red line) and MPO (blue line) (n = 3 mice for MPOA, n = 6 mice for VMHvl). P: comparison of dynamic velocity for similar behavior between VMHvl and MPOA (reproduced from Figure 6F, 6K) (**p<0.005,***p<0.001) (n = 3 mice for MPOA, n = 6 mice for VMHvl)

7 Supplementary Video 1: neural trajectories and flow fields of VMHvl mouse 1 alongside behavior
Related to Figure 3
Video indicates the movement of neural trajectories in a low dimensional neural state space with inferred flow fields from dynamical systems overlayed. Behavior of the resident and intruder is shown on the left.

8 Supplementary Video 2: neural trajectories and dynamics landscapes of VMHvl mouse 1
Related to Figure 3
Video indicates the movement of neural trajectories in a low dimensional neural state space with inferred dynamics landscape from dynamical systems overlayed.

## Figures and Tables

**Table:** KEY RESOURCES TABLE

REAGENT or RESOURCE	SOURCE	IDENTIFIER
Deposited Data		
neural imaging data from VMHvl and MPOA Esr1 neurons	Karigo et al., Nature 2021	10.1038/s41586–020-2995–0
neural imaging data from VMHvl Esr 1 neurons	Yang et al., Nature 2022	10.1038/s41586–022-05057–6
neural imaging data from VMHvl Esr1 neurons	Remedios et al., 2017	10.1038/nature23885
Software and Algorithms		
Matlab 2020a	Mathworks	
Python	Python Software Foundation	

## Highlights

- Dynamical system analysis reveals a line attractor in VMHvl^Esr1^ neuronal activity (83)
- The line attractor represents a neural correlate of aggressive escalation (75)
- Individual differences in aggression are correlated with line attractor properties (84)
- MPOA^Esr1^ utilizes rotational not line attractor dynamics to encode mating behavior (84)
