# Supplementary figures and images for: An approximate line attractor in the hypothalamus encodes an aggressive state
Source: Cell. Author manuscript; Available in PMC 2023 Mar 7. (PMC9990527; doi:10.1016/j.cell.2022.11.027)

# Supplementary Figure 1

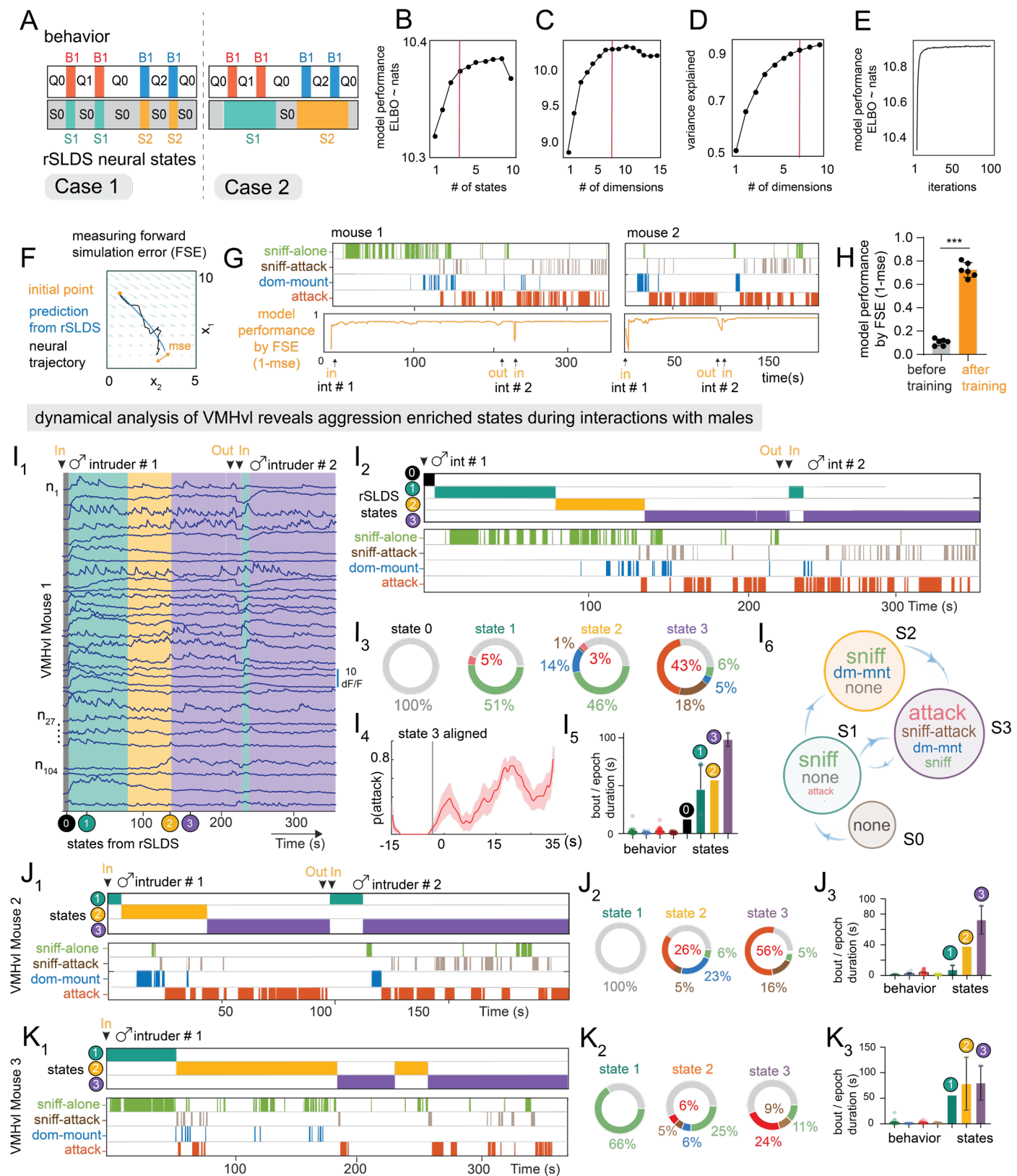

Supplement: 1 — Supplementary Figure 1: Unsupervised discovery of aggression-enriched states in VMHvl Related to Figure 2 A: types of neural states identified by rSLDS. B1, B2: behaviors; Q0, Q1: periods of quiescence between behavior bouts; S0,S1,S2: rSLDS states. Case 1: rSLDS states cannot distinguish behavior vs internal states. Case 2: rSLDS reflects internal state-encoding due to persistence during behavioral quiescence. B: optimization of number of rSLDS states in example VMHvl mouse 1. Model performance is measured as ELBO (see methods). C: same as B, but for dimensionality. D: variance explained by dimension chosen in C. E: convergence of model performance. F: creation of a bounded model performance metric (forward simulation error, FSE, see methods). G: FSE for VMHvl mouse 1 & 2. H: average model performance (FSE) before and after training (n = 6 mice,***p<0.001).: I1: rSLDS states in VMHvl mouse 1. I2: comparison of rSLDS states with behaviors. I3: behavioral composition of rSLDS states. State 3 possesses the highest amount of attack behavior across mice (see panel J, K). I4: probability of attack aligned to the onset of state 3 (n = 6 mice). I5: timescale of behavior bouts and discovered states epochs. I6: state transition diagram from empirical transition probabilities. J: Same as F2, F3, F5 but for VMHvl mouse 2. K: Same as F2, F3, F5 but for VMHvl mouse 3. [file NIHMS1861402-supplement-1.pdf]

## properties of integration dimension in VMHvl during interactions with males

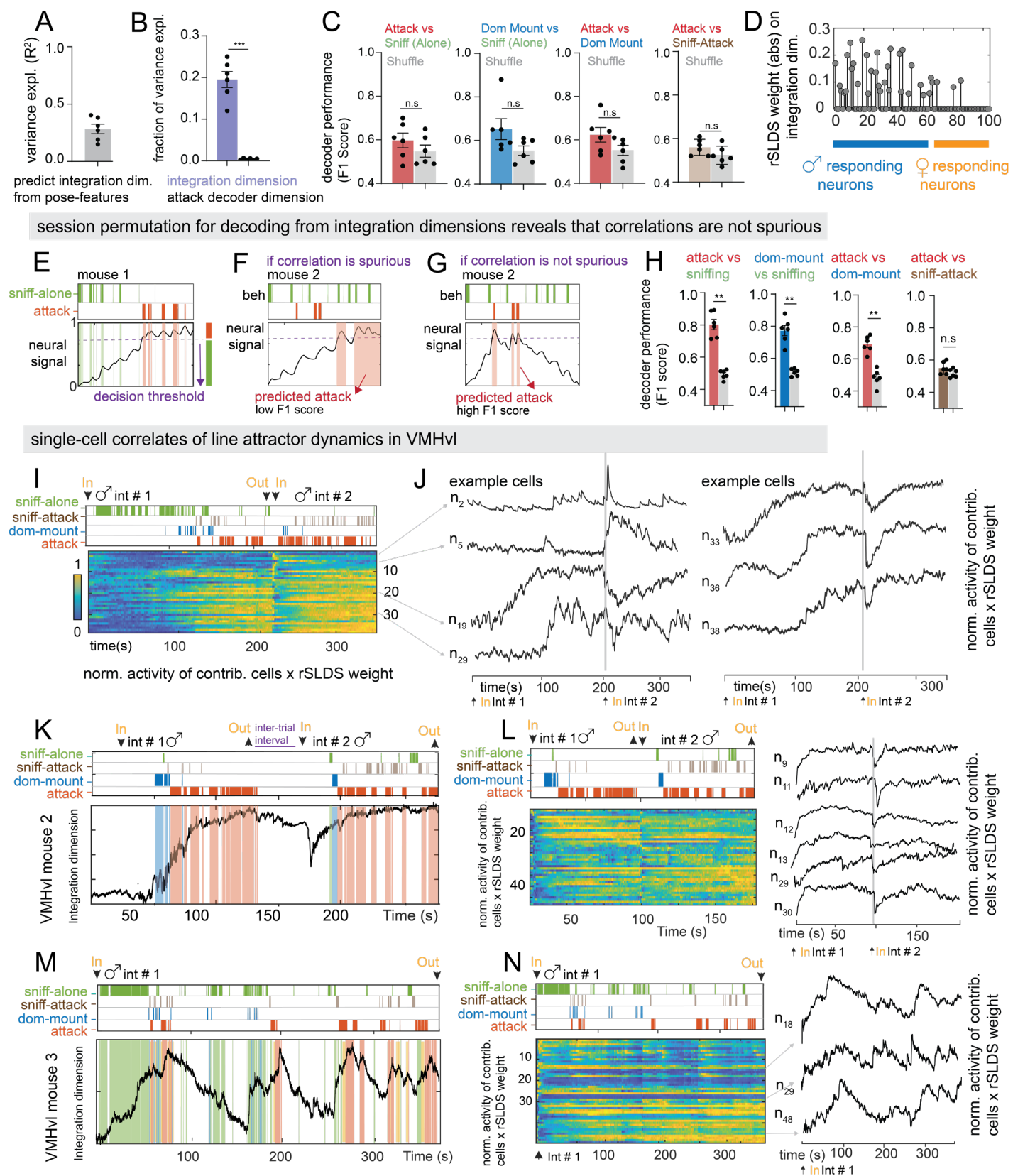

Supplement: 2 — Supplementary Figure 2: Characterization of aggression-integration dimension Related to Figure 2 A: variance explained by a generalized linear model trained to predict integration dimension from pose-features including distance between mice, facing angle, speed, acceleration, and velocity of resident mouse (mean: 0.28 ± 0.04 R2, n = 6 mice). B: fraction of overall variance explained by integration dimension (purple) compared to variance explained by decoder dimension trained to distinguish attack from sniff bouts (integration dimension mean: 19.5%± 1.9%, attack decoder mean: 0.3% ± 0.1%, n= 6 mice, ***p<0.001). C: decoding behaviors from non-integration dimensions (average across dimensions, n = 6 mice). D: absolute rSLDS weight on integration dimension of VMHvl mouse 1 (cell number on x-axis), sorted by choice probability values for male vs female intruder encounter. E-G: paradigm to account for spurious correlations: decoding threshold obtained using integration dimension of mouse 1 (E, purple line) is used on integration dimension from mouse 2 (F). Spurious correlations lead to low F1 scores (F) while true correlations retain high F1 scores (G). H: decoding behaviors using paradigm described above (**p < 0.005, n = 6 mice) I: normalized activity of neurons times rSLDS weight for cells with significant weights for integration dimension of VMHvl mouse 1. J: example cells from I. K: integration dimension in VMHvl mouse 2. L: same as I for VMHvl mouse 2. M,N: Same as K,L for VMHvl mouse 3. [file NIHMS1861402-supplement-2.pdf]

# dynamical analysis of VMHvl in mating behavior

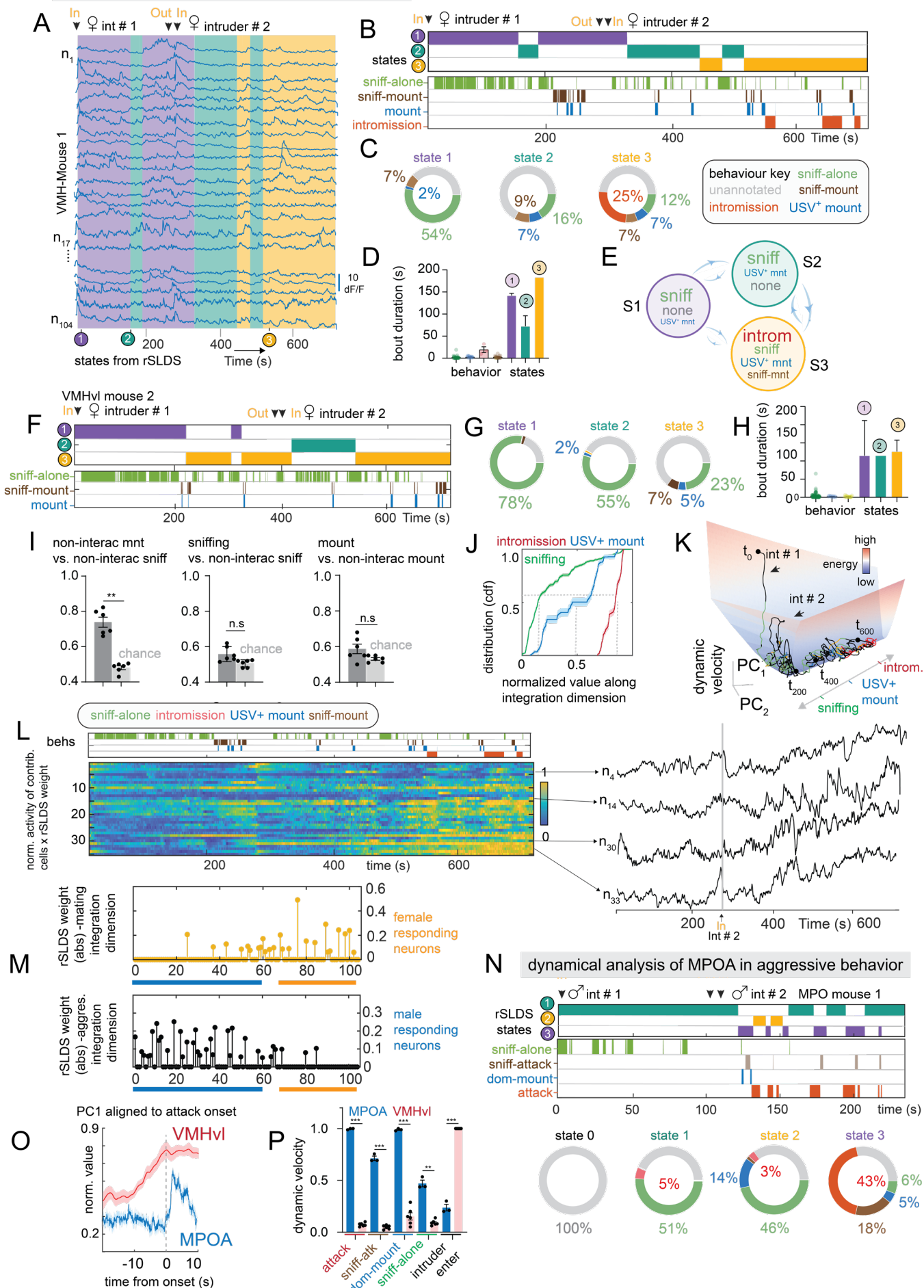

Supplementary Figure 6

Supplement: 6 — Supplementary Figure 6: Dynamical analysis of VMHvl activity in mating behavior and MPOA activity in aggression. Related to Figure 6 A: rSLDS states in VMHvl mouse 1 during interactions with female intruders. B: comparison of rSLDS states with behaviors. C: behavioral composition of rSLDS states. State 3 possesses the highest amount of mating behavior across mice (see panel H). D: timescale of behavior bouts and state epochs. E: state transition diagram from empirical transition probabilities. F-H: Same as B-D for VMHvl mouse 2. This mouse did not achieve intromission. I: decoding behaviors from integration dimension (**p<0.005). ). J: empirical cumulative distribution of value of integration dimension (normalized) for various behaviors. K: dynamics velocity landscape showing a progression of mating behavior along the trough for VMHvl mouse 1. L: normalized activity times rSLDS weight for cells contributing significantly to integration dimension of VMHvl mouse 1. M: absolute rSLDS weight on integration dimension of VMHvl mouse 1 during mating behavior (top, yellow dots) and aggression (bottom, black dots) sorted by choice probability values for male vs female intruder encounter. N: top: state and behavior raster for MPOA mouse 1 during aggressive behavior. State 3 is aligned closely to the onset of attack bouts, bottom: behavioral composition of discovered states. O: behavior triggered average of principal component 1 in VMHvl (red line) and MPO (blue line) (n = 3 mice for MPOA, n = 6 mice for VMHvl). P: comparison of dynamic velocity for similar behavior between VMHvl and MPOA (reproduced from Figure 6F, 6K) (**p<0.005,***p<0.001) (n = 3 mice for MPOA, n = 6 mice for VMHvl) [file NIHMS1861402-supplement-6.pdf]
