# Supplementary material for: An approximate line attractor in the hypothalamus encodes an aggressive state
Source: Cell. Author manuscript; Available in PMC 2023 Mar 7. (PMC9990527; doi:10.1016/j.cell.2022.11.027)

Supplementary Figure 3

dynamics of aggression integration dimension during interactions with females

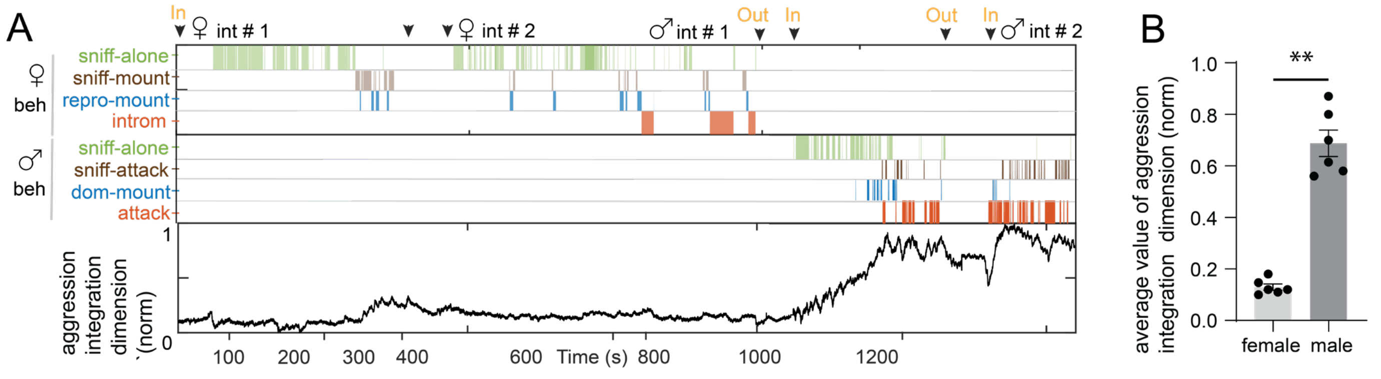

rSLDS discovers line attractor dynamics in VMHvl even in the absence of external tracking inputs

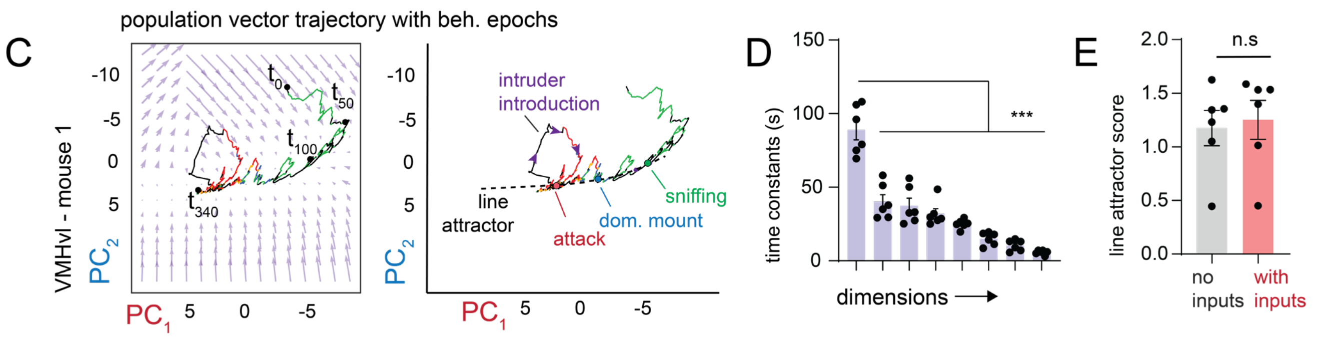

tracking features used in rSLDS are not predictive of behavioral annotations

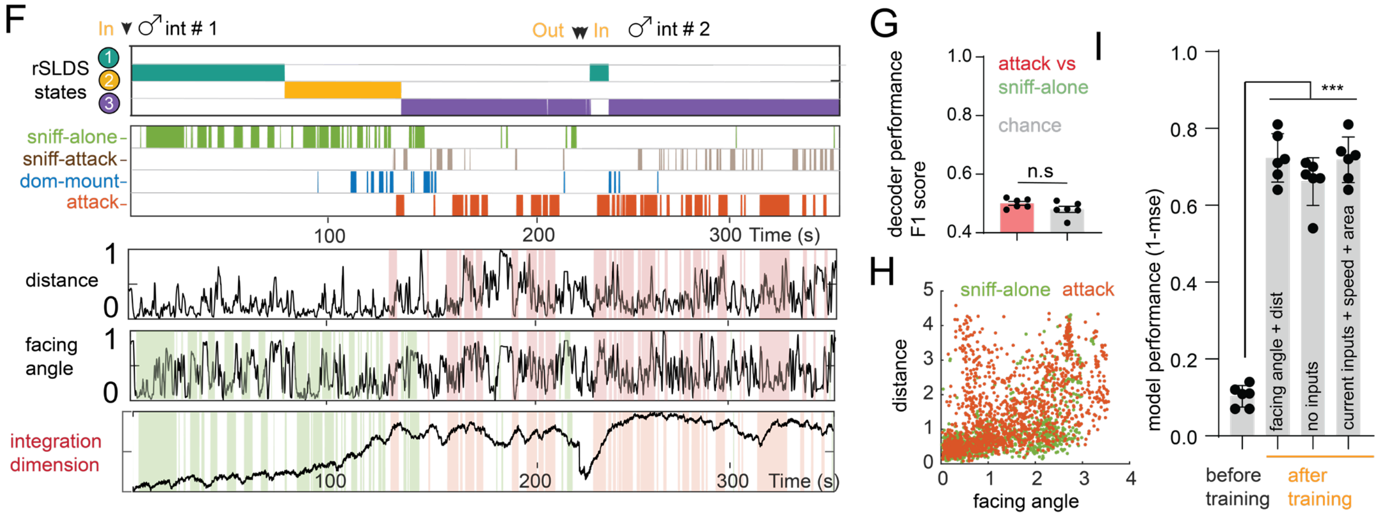

Supplement: 3 — Supplementary Figure 3: Characterization of aggression-integration dimension and dependence on tracking feature based external inputs. Related to Figure 2 A: aggression integration dimension in female and male trials in VMHvl Mouse 1. B: mean projection of neural activity from female vs male trials onto the aggression integration dimension (n = 6 mice, **p<0.005). C: low dimensional dynamics and flow field from model with no behavioral inputs included with line attractor highlighted. D: time constants from the fit dynamical system (n = 6 mice). E: line attractor score for VMHvl models without input. F: tracking features used in rSLDS shown alongside discovered states and integration dimension in VMHvl mouse 1. G: performance of decoder used to separate attack frames from sniff-alone frames using the distance between mice and facing angle of the resident. H: scatter plot of distance between mice and facing angle of resident. I: model performance (1-FSE) for different types of external inputs (n = 6 mice); current inputs = distance between animals, facing angle of resident. (***p<0.001). [file NIHMS1861402-supplement-3.pdf]
