# Supplementary material for: An approximate line attractor in the hypothalamus encodes an aggressive state
Source: Cell. Author manuscript; Available in PMC 2023 Mar 7. (PMC9990527; doi:10.1016/j.cell.2022.11.027)

Supplementary Figure 4

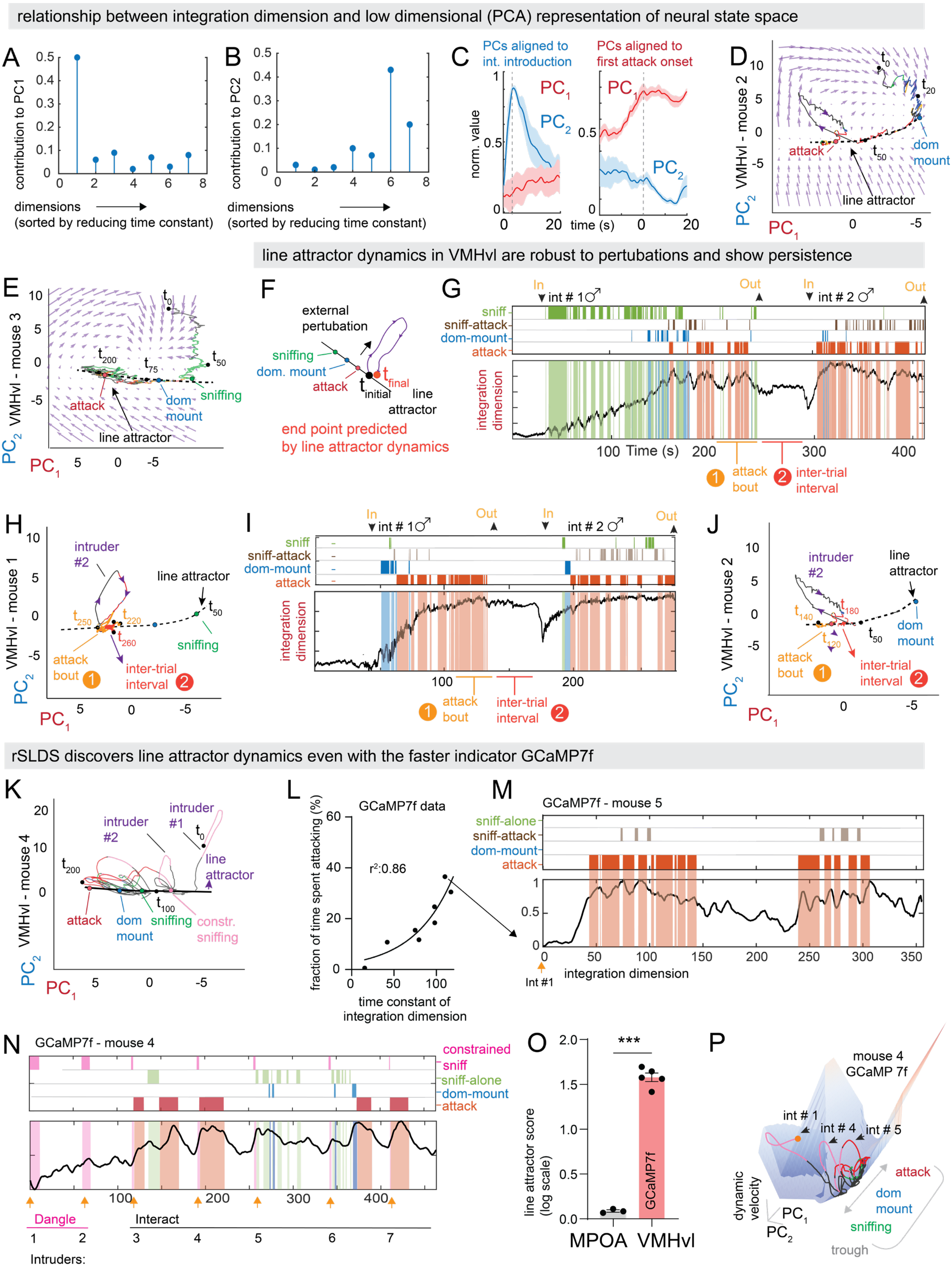

Supplement: 4 — Supplementary Figure 4: Properties of line attractor dynamics in VMHvl. Related to Figure 3 A,B: absolute PCA weights of PC1(A) and PC2(B) on dimensions of dynamical system sorted by decreasing time constant in VMHvl mouse 1. C: behavior triggered average of top two principal components aligned to introduction of first intruder or first attack onset (n = 6 mice). D,E: low dimensional dynamics and flow field showing line attractor dynamics for VMHvl mouse 2 and mouse 3 with line attractor highlighted. F: schematic showing how perturbations orthogonal to a line attractor do not alter the position of the system. G: integration dimension in VMHvl mouse 1 (reproduced from Fig 2B) with attack bout (1) and inter-trial interval (2) highlighted. H: neural state space with line attractor highlighted in VMHvl mouse 1, showing the persistence of activity during the inter-trial interval shown in G. The introduction of intruder #2 acts as an orthogonal perturbation and activity returns to the same point along the attractor. I,J: Same as G,H for VMHvl mouse 2. K: neural state space with line attractor highlighted in VMHvl mouse 4. The introduction of intruder #2 occurs earlier in the trial when the animal displays sniffing behavior but results in a similar perturbation as above. L: relationship between fraction of time spent attack vs time constant of integration for animals with GCaMP7f recordings (n= 8 mice). M: integration dimension in VMHvl mouse 5 (GCaMP 7f) shows the same persistence and slow decay of activity. N: same as M for VMHvl mouse 4 (GCaMP 7f). O: line attractor score for mice with GCaMP7f recordings (***p<0.001). P: dynamics landscape for VMHvl mouse 4 (GCaMP 7f) showing a trough shaped landscape. [file NIHMS1861402-supplement-4.pdf]
