# Supplementary material for: An approximate line attractor in the hypothalamus encodes an aggressive state
Source: Cell. Author manuscript; Available in PMC 2023 Mar 7. (PMC9990527; doi:10.1016/j.cell.2022.11.027)
Supplement: 5 — Supplementary Figure 5: Mating enriched states and rotational dynamics in MPOA. Related to Figure 4 A: rSLDS states in MPOA mouse 1. B: comparison of rSLDS states with behavior in MPOA mouse 1 for period from t = 600s to t = 700s. C: behavioral composition of rSLDS states. D: probability of intromission and USV+ mounting aligned to the onset of state 2 and state 3 (also see panel I, J, n = 3 mice). E: timescale of behavioral bouts and states epochs. F: Reproduced from Figure 4D but with state-specific inferred flow-field colors. G: state transition diagram from empirically calculated transition probabilities. H: state and behavior raster for MPOA mouse 1 for entire recording. I1: same as H for MPOA mouse 2, selected mating bouts highlighted. I2: behavioral composition of rSLDS states (bottom). I3: timescale of behavioral bouts and states epochs. J1–3: same as I1–3 for MPOA mouse 3, selected mating bouts highlighted. K: rotational trajectories for 3 mating episodes in MPOA mouse 1. L: same as K, for mating bouts highlighted in highlighted in I1 for MPOA mouse 2. M: same as K, for mating bouts highlighted in highlighted in J1 for MPOA mouse 3. [file NIHMS1861402-supplement-5.pdf]

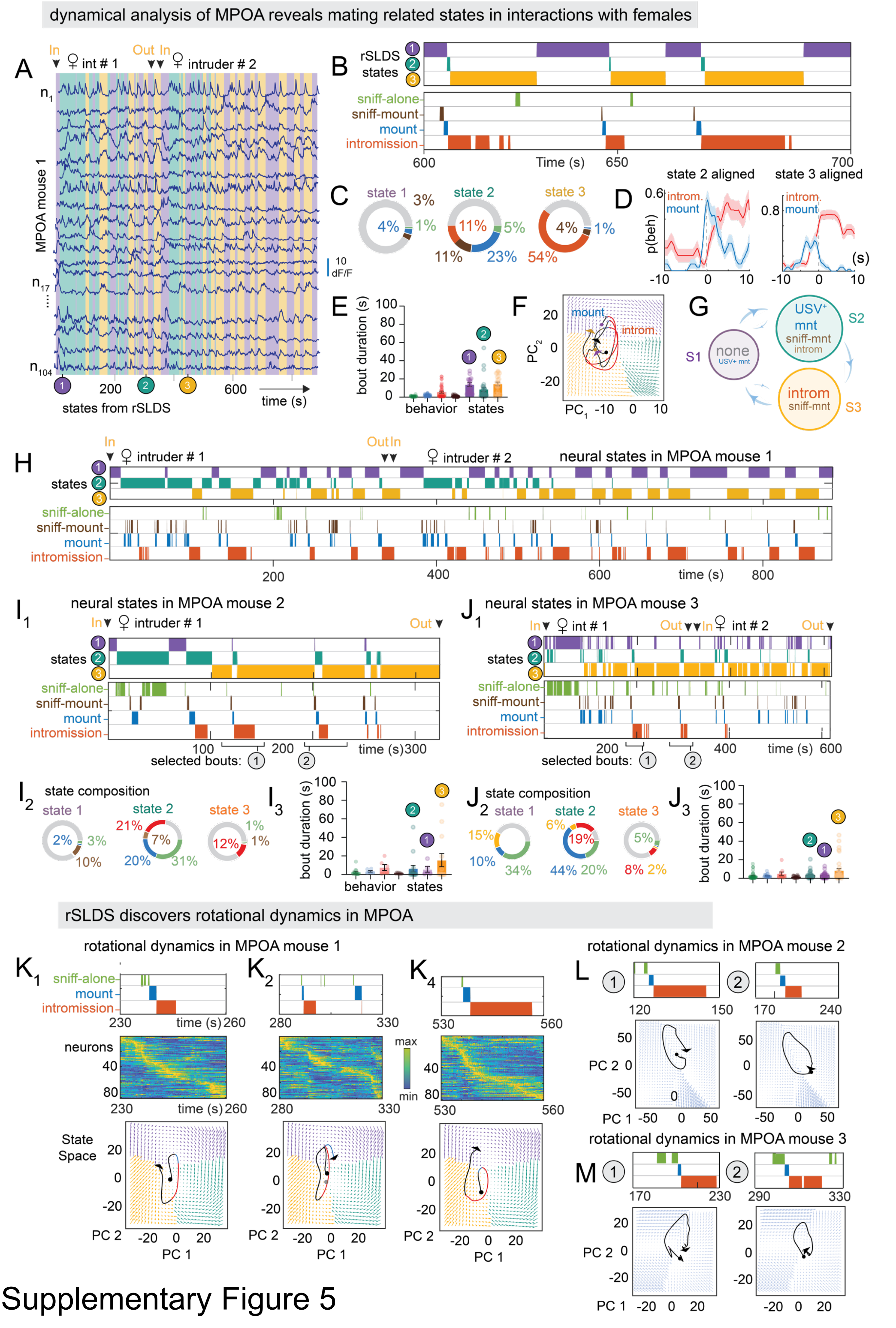

Supplementary Figure 5
